# Genetic aspects of adolescent idiopathic scoliosis in a family with multiple affected members: a research article

**DOI:** 10.1186/1748-7161-5-7

**Published:** 2010-04-07

**Authors:** Marcelo Wajchenberg, Monize Lazar, Natale Cavaçana, Delio Eulalio Martins, Luciana Licinio, Eduardo Barros Puertas, Elcio Landim, Mayana Zatz, Akira Ishida

**Affiliations:** 1 Universidade Federal de São Paulo. Rua Borges Lagoa 783, 5th floor - Vila Clementino - Sao Paulo ZIP 04038-032, Brazil; 2 Universidade de São Paulo, Rua do Matao, 277 Sala 211 - Cidade Universitaria - Butanta - Sao Paulo - ZIP 05508-090, Brazil

## Abstract

**Background:**

The etiology of idiopathic scoliosis remains unknown and different factors have been suggested as causal. Hereditary factors can also determine the etiology of the disease; however, the pattern of inheritance remains unknown. Autosomal dominant, X-linked and multifactorial patterns of inheritances have been reported. Other studies have suggested possible chromosome regions related to the etiology of idiopathic scoliosis. We report the genetic aspects of and investigate chromosome regions for adolescent idiopathic scoliosis in a Brazilian family.

**Methods:**

Evaluation of 57 family members, distributed over 4 generations of a Brazilian family, with 9 carriers of adolescent idiopathic scoliosis. The proband presented a scoliotic curve of 75 degrees, as determined by the Cobb method. Genomic DNA from family members was genotyped.

**Results:**

Locating a chromosome region linked to adolescent idiopathic scoliosis was not possible in the family studied.

**Conclusion:**

While it was not possible to determine a chromosome region responsible for adolescent idiopathic scoliosis by investigation of genetic linkage using microsatellites markers during analysis of four generations of a Brazilian family with multiple affected members, analysis including other types of genomic variations, like single nucleotide polymorphisms (SNPs) could contribute to the continuity of this study.

## Background

Idiopathic scoliosis is a structural lateral curvature of the spine with a rotatory component deviation in an otherwise healthy individual. These individuals present no known neurological, muscular disorders or other diseases [1] Radiography exam shows no vertebral alterations, while present with curves of more than 10°, as determined by the Cobb method [1]. Idiopathic scoliosis is one of the most frequent deformity involving the spine, with reports of its incidence in populations worldwide from 0.5% to 10%. Scoliosis progresses during the growth phase and can be classified into three categories according to the age at which the deformity is detected: infant, prior to three years of age; juvenile, between three and 10 years-old (or at the onset of puberty); and adolescent, when it appears after 10 years of age or after the onset of puberty [2].

Lonstein (1994) reported that the prevalence of idiopathic scoliosis in radiographic studies in school populations varied between 0.3 and 15.3%; however, considering only curves greater than 10°, the rates decreased to values between 1.5 and 3%. In curves greater than 20°, prevalence occurred between 0.3 and 0.5% and in curves greater than 30° the rate was between 0.2 and 0.3% [2].

The pathogenesis of idiopathic scoliosis remains unknown and different factors have been suggested as causal. Among these, the following should be highlighted: deviation from the standard growth pattern, neuromuscular or conjunctive tissue alterations, asymmetric growth of the limbs and trunk, alterations in the sagittal configuration of the spine; and environmental factors [2-5].

Hereditary factors can also determine the etiology of the disease; however, the pattern of inheritance remains unknown. Autosomal dominant, X-linked and multifactorial patterns of inheritances have been reported [1-3,5-8]. Segregation analysis has suggested a single gene as major determinant of idiopathic scoliosis in patients with curves equal to or greater than 11° (Axenovich et al. 1999). Other studies have suggested possible chromosome regions related to the etiology of idiopathic scoliosis, including a genetic locus for adolescent idiopathic scoliosis linked to chromosome 19p13.3., considering affected members as those individuals with curves greater than 10°, without reference to the presence of consanguinity [1,3,7-9].

The objective of this study is to determine a specific chromosome region related to idiopathic scoliosis in a family with multiple affected members and a high rate of consanguinity in a small town in the outback of the State of Paraiba, northeastern Brazil.

## Methods

We investigated 57 members of a four-generation family of Brazilian ancestry. Each family member who agreed to participate was clinically examined. Characterization of family members included sex, age and physical and ethnical characteristics, such as biotype, as well as the degree of deformity determined by the Cobb method using standing anteroposterior spinal radiographs. All of the members were skeletally mature at the time of assessment.

The criteria for selecting the family included clinical and radiological diagnosis of idiopathic scoliosis in at least seven family members with scoliotic curves greater than 15° according to the Cobb method. The family selected for this research was chosen from a previous study involving 100 families of patients with adolescent idiopathic scoliosis, conducted by Wajchenberg et al (2005) [5].

The proband underwent surgery for adolescent idiopathic scoliosis in 2002, due to a curve of 75°. An investigation into the patient's family was conducted by means of interviews and affected members were verified in Sao Paulo and in the home town of the patient's family, Sao Jose de Piranhas, in the outback of the State of Paraiba.

Family members were divided into three groups: affected individuals, unaffected and suspected disease. Affected members were classified as those presenting clinical and radiological (curvature greater than 15°) signs characteristic of the disease. Individuals suspected of having the disease showed clinical and radiological signs suggestive of idiopathic scoliosis (curvature between 5 and 14°), while unaffected individuals showed no signs of the disease. The individuals were submitted to peripheral blood collection for genomic DNA extraction and subsequent linkage analysis, using microsatellite markers from the ABI PRISM^® ^Linkage Mapping Set, Version 2, (Applied Biosystems). The MERLIN program was used for statistical analyses in order to calculate the lods (logarithm of the odds ratios) scores, based on the consideration that the family presented an autosomal dominant pattern of inheritance with incomplete penetrance estimated in 35% and allele frequency of 0.01%. Informed consent was obtained from all subjects.

## Results

**Figure 1:**
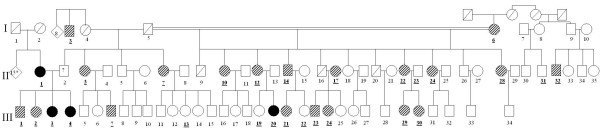
**Heredogram of the paternal lineage of the proband (III-3)**.

**Figure 2:**
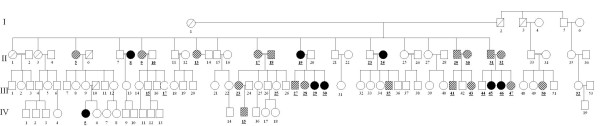
**Heredogram of the maternal lineage of the proband (III-29)**.

Evaluation of familial history of the individuals studied revealed an autosomal dominant pattern of transmission (Figures 1 and 2), with 9 affected members, 12 unaffected members and 36 members showing some characteristics of the disease, classified as suggestive of idiopathic scoliosis.

Consanguinity was determined and in the heredogram constructed, each family member was assigned a bold underlined number. Linkage analysis was unable to determine a specific chromosome region related to the disease in this study according to the tables (additional file 1) and graphics (additional file 2) from chromosomes 1 to 22.

## Discussion

The etiology and pattern of inheritance for the transmission of idiopathic scoliosis remain unknown, though studies of families with multiple affected members suggest that a single gene or limited number of genes are linked to the disease. Recent studies have used an autosomal dominant pattern of transmission, with incomplete penetrance and variable expressivity, to conduct linkage studies [1,3,8,9].

This pattern was also observed in the family studied in this study; however, the high level of consanguinity observed should be highlighted, as well as the fact that the family originates from a small town of approximately 15,000 inhabitants, for this reason, we also have done a linkage analysis considering an autosomal recessive pattern of inheritance and allele frequency of 0.01%. Nevertheless, we did not find any specific chromosome region associated with the disease.

The criteria for characterization of an individual as affected by the disease in the literature includes the presence of scoliosis equal to or greater than 10° as determined by the Cobb method, with vertebral rotation observed in radiographs realized in the frontal position in orthostasis [1-3,5-9].

Axenovich et al (1999) conducted segregation analysis on 101 families with idiopathic scoliosis patients and observed a monogenic, dominant pattern of inheritance, using a diallelic model, considering individuals with curvature greater than 11° as affected [6]. Wise et al (2000) studied families whose probands presented curves equal to or greater than 50° or who required corrective surgery. Among the families analyzed in previous works, expressive variation was observed in the spinal curve values of affected patients, which suggests the possible influence of other genetic and environmental factors [8]. In the present study, we analyzed affected individuals with a physical exam compatible with idiopathic scoliosis and radiographs indicating curvature equal to or greater than 15°. Individuals suspected of having the disease showed some clinical sign and radiographs suggestive of idiopathic scoliosis, though a curvature greater than 10° was not considered obligatory. Thus, it was possible to construct a heredogram with an autosomal dominant characteristic. The family studied showed characteristics typical of idiopathic scoliosis; notably the individuals were Caucasian and the female members were predominantly affected (100%).

Since 2000, familial studies have been conducted in an attempt to map the chromosome regions responsible for idiopathic scoliosis. Wise et al (2000) suggested that regions on chromosomes 6, 10 and 18 were related to idiopathic scoliosis [8]. Salehi et al (2002) investigated three generations of one family of Italian origin with 11 affected members who presented curves between 10 and 20° and an autosomal dominant pattern of inheritance with complete penetrance [7]. Their study mapped a region of approximately 20 cM on chromosome 17p11 linked to idiopathic scoliosis. In the same year, Chan et al (2002) identified a region of 5.2 cM in the region 19p13 and reported a secondary candidate region on chromosome 2, while studying seven Chinese families with an autosomal dominant pattern of inheritance and probands with curves between 20 and 55° that required the use of a brace or surgical treatment [9]. In 2005, Alden et al also reported a region on chromosome 19 with potential links to idiopathic scoliosis, when studying 1198 individuals from 202 families that presented at least 2 affected members with curves equal to or greater than 11°. However, correlation with region 19p13 only occurred when analyzing families with probands with curvature equal to or greater than 30° [3]. Ocaka et al (2008) studied 25 unrelated multiplex adolescent idiopathic scoliosis families [1]. Of these, 24 families were Caucasian of British descent, and one was of African-Caribbean origin. The authors considered families with at least three affected members who had curves equal to or greater than 10°, with rotation. The probands had curves that required orthopedic treatment, varying 20 and 55°. An autosomal dominant pattern of inheritance with penetrance estimated at 80% was verified. The authors reported two chromosome regions potentially linked to adolescent idiopathic scoliosis on chromosomes 9p34 and 17q25.

## Conclusion

In the family analyzed in this study, it was not possible to determine a chromosome region responsible for idiopathic scoliosis by investigation of genetic linkage. One explanation for the inconclusive linkage analysis is the possibility that the disease is not monogenic; it is also possible that the genes involved do not contribute equally to determining the phenotype in these patients.

Nevertheless, we continue to believe in the importance of research involving families with numerous members affected by idiopathic scoliosis. Our efforts now consist in study the commons variations in the genome, like single nucleotide polymorphisms (SNPs), using microarray SNPs experiment, with the aim of determining chromosome regions linked to the disease.

## Competing interests

The authors declare that they have no competing interests.

## Authors' contributions

MW carried out the conception and design of the study as well as the acquisition, analysis and interpretation of data. MZ conceived the study and participated in its design and coordination and helped to draft the manuscript. ML carried out the molecular genetic studies. NC carried out the molecular genetic studies. EBP conceived the study and participated in its design and coordination and helped to draft the manuscript. DEM carried out critical revision of the study for important intellectual content. LL carried out the molecular genetic studies. EL gave final approval of the version to be published. All authors read and approved the final manuscript.

## Supplementary Material

Additional file 1 Tables presenting the estimated multipoint LOD score at chromosomes 1 to 22. Click here for file

Additional file 2 Graphics presenting the parametric analysis for dominant model at chromosomes 1 to 22. Click here for file
