# Supplementary material for: Genetic aspects of adolescent idiopathic scoliosis in a family with multiple affected members: a research article
Source: Scoliosis. 2010 Apr 7;5:7. doi: 10.1186/1748-7161-5-7 (PMC2873229; doi:10.1186/1748-7161-5-7)
Supplement: Additional file 1 — Tables presenting the estimated multipoint LOD score at chromosomes 1 to 22. [file 1748-7161-5-7-S1.PDF]

Table 01. Estimated multipoint LOD score at chromosome 01

CHR	MARKER	POSITION (cM)	LOD	ALPHA	HLOD
1	D1S468	4.220	-0.002	0.000	0.000
1	D1S214	14.040	-0.004	0.000	0.000
1	D1S450	20.610	-0.005	0.000	0.000
1	D1S2667	24.680	-0.004	0.000	0.000
1	D1S2697	37.050	-1.105	0.000	0.000
1	D1S199	45.330	-1.104	0.000	0.000
1	D1S234	55.100	-1.105	0.000	0.000
1	D1S255	65.470	-1.102	0.000	0.000
1	D1S2797	75.660	-0.930	0.000	0.000
1	D1S2890	85.680	-1.105	0.000	0.000
1	D1S230	95.310	-0.929	0.000	0.000
1	D1S2841	106.450	-1.105	0.000	0.000
1	D1S207	113.690	-1.105	0.000	0.000
1	D1S2868	126.160	-0.952	0.000	0.000
1	D1S206	134.200	-1.105	0.000	0.000
1	D1S2726	144.380	-1.105	0.000	0.000
1	D1S252	150.270	-0.561	0.000	0.000
1	D1S498	155.890	-0.230	0.000	0.000
1	D1S484	169.680	0.285	1.000	0.285
1	D1S2878	177.860	-0.185	0.000	0.000
1	D1S196	181.490	-1.102	0.000	0.000
1	D1S218	191.520	-1.102	0.000	0.000
1	D1S238	202.730	-1.102	0.000	0.000
1	D1S413	212.440	-0.868	0.000	0.000
1	D1S249	220.650	-1.105	0.000	0.000
1	D1S425	231.110	-1.105	0.000	0.000
1	D1S213	242.340	-1.105	0.000	0.000
1	D1S2800	252.120	-1.105	0.000	0.000
1	D1S2785	266.270	0.287	1.000	0.287
1	D1S2842	273.460	0.280	1.000	0.280
1	D1S2836	285.750	0.287	1.000	0.287

Chr: Chromosome; LOD: estimated multipoint LOD score at a particular location;
ALPHA: estimate proportion of linked families (since there is only one informative family in this sample, the proportion will always be 0.000 or 1.000);
HLOD: maximum heterogeneity LOD score

Table 02. Estimated multipoint LOD score at chromosome 02

CHR	MARKER	POSITION (cM)	LOD	ALPHA	HLOD
2	D2S319	7.600	-0.107	0.000	0.000
2	D2S2211	15.610	0.280	1.000	0.280
2	D2S162	20.030	0.081	1.000	0.081
2	D2S168	27.060	-1.102	0.000	0.000
2	D2S305	38.870	-1.102	0.000	0.000
2	D2S165	47.430	-1.102	0.000	0.000
2	D2S367	54.960	-1.102	0.000	0.000
2	D2S2259	64.290	-1.102	0.000	0.000
2	D2S391	70.310	-1.102	0.000	0.000
2	D2S337	80.690	-1.102	0.000	0.000
2	D2S2368	85.480	-1.105	0.000	0.000
2	D2S286	94.030	-0.021	0.000	0.000
2	D2S2333	103.160	0.269	1.000	0.269
2	D2S2216	111.210	0.280	1.000	0.280
2	D2S160	122.960	0.269	1.000	0.269
2	D2S347	131.510	0.022	1.000	0.022
2	D2S112	141.620	-1.105	0.000	0.000
2	D2S151	152.040	-0.883	0.000	0.000
2	D2S142	161.260	-0.766	0.000	0.000
2	D2S2330	169.410	-0.891	0.000	0.000
2	D2S335	175.910	-1.008	0.000	0.000
2	D2S364	186.210	-1.102	0.000	0.000
2	D2S117	194.450	-0.184	0.000	0.000
2	D2S325	204.530	0.118	1.000	0.118
2	D2S2382	213.490	0.287	1.000	0.287
2	D2S126	221.130	0.287	1.000	0.287
2	D2S396	232.900	0.280	1.000	0.280
2	D2S206	240.790	-0.004	0.000	0.000
2	D2S338	250.540	0.280	1.000	0.280
2	D2S125	260.630	0.212	1.000	0.212

Chr: Chromosome; LOD: estimated multipoint LOD score at a particular location;
ALPHA: estimate proportion of linked families (since there is only one informative family in this sample, the proportion will always be 0.000 or 1.000);
HLOD: maximum heterogeneity LOD score

Table 03. Estimated multipoint LOD score at chromosome 03

CHR	MARKER	POSITION (cM)	LOD	ALPHA	HLOD
3	D3S1297	8.310	0.287	1.000	0.287
3	D3S1304	22.330	0.271	1.000	0.271
3	D3S1263	36.100	0.287	1.000	0.287
3	D3S2338	42.100	0.287	1.000	0.287
3	D3S1266	52.600	0.287	1.000	0.287
3	D3S1277	61.520	0.287	1.000	0.287
3	D3S1289	71.410	0.287	1.000	0.287
3	D3S1300	80.320	0.287	1.000	0.287
3	D3S1285	104.410	0.287	1.000	0.287
3	D3S1566	109.220	0.286	1.000	0.286
3	D3S3681	111.110	0.287	1.000	0.287
3	D3S1271	117.760	0.287	1.000	0.287
3	D3S1278	129.730	-1.102	0.000	0.000
3	D3S1267	139.120	-1.102	0.000	0.000
3	D3S1292	146.600	-1.102	0.000	0.000
3	D3S1569	158.380	-1.104	0.000	0.000
3	D3S1279	169.900	0.280	1.000	0.280
3	D3S1614	177.750	0.248	1.000	0.248
3	D3S1565	186.040	0.280	1.000	0.280
3	D3S1262	201.140	0.260	1.000	0.260
3	D3S1580	207.730	0.280	1.000	0.280
3	D3S1601	214.450	0.279	1.000	0.279
3	D3S1311	224.880	0.287	1.000	0.287

Chr: Chromosome; LOD: estimated multipoint LOD score at a particular location;
ALPHA: estimate proportion of linked families (since there is only one informative family in this sample, the proportion will always be 0.000 or 1.000);
HLOD: maximum heterogeneity LOD score

Table 04. Estimated multipoint LOD score at chromosome 04

CHR	MARKER	POSITION (cM)	LOD	ALPHA	HLOD
4	D4S412	4.740	-1.105	0.000	0.000
4	D4S2935	13.960	-0.620	0.000	0.000
4	D4S403	25.900	-0.527	0.000	0.000
4	D4S419	33.420	-0.601	0.000	0.000
4	D4S391	43.590	-1.105	0.000	0.000
4	D4S405	56.950	0.280	1.000	0.280
4	D4S1592	69.530	0.234	1.000	0.234
4	D4S392	78.970	0.230	1.000	0.230
4	D4S2964	88.350	0.250	1.000	0.250
4	D4S1534	95.090	0.280	1.000	0.280
4	D4S414	100.750	0.280	1.000	0.280
4	D4S1572	107.950	0.269	1.000	0.269
4	D4S406	117.060	0.285	1.000	0.285
4	D4S402	124.450	0.270	1.000	0.270
4	D4S1575	132.050	0.159	1.000	0.159
4	D4S424	144.560	-0.089	0.000	0.000
4	D4S413	157.990	-1.103	0.000	0.000
4	D4S1597	169.420	-0.174	0.000	0.000
4	D4S1539	176.190	0.134	1.000	0.134
4	D4S415	181.360	0.280	1.000	0.280
4	D4S1535	195.060	0.182	1.000	0.182
4	D4S426	206.980	0.060	1.000	0.060

Chr: Chromosome; LOD: estimated multipoint LOD score at a particular location;
ALPHA: estimate proportion of linked families (since there is only one informative family in this sample, the proportion will always be 0.000 or 1.000);
HLOD: maximum heterogeneity LOD score

Table 05. Estimated multipoint LOD score at chromosome 05

CHR	MARKER	POSITION (cM)	LOD	ALPHA	HLOD
5	D5S1981	1.720	-0.414	0.000	0.000
5	D5S406	11.850	-1.104	0.000	0.000
5	D5S630	19.670	-0.220	0.000	0.000
5	D5S416	28.760	0.067	1.000	0.067
5	D5S419	39.990	0.280	1.000	0.280
5	D5S426	51.990	-0.131	0.000	0.000
5	D5S418	58.550	-1.102	0.000	0.000
5	D5S407	64.670	-1.102	0.000	0.000
5	D5S647	74.070	-1.102	0.000	0.000
5	D5S424	81.950	-1.105	0.000	0.000
5	D5S641	92.380	-0.966	0.000	0.000
5	D5S428	95.400	-0.966	0.000	0.000
5	D5S644	104.760	-1.096	0.000	0.000
5	D5S433	111.970	-1.102	0.000	0.000
5	D5S2027	119.500	-1.102	0.000	0.000
5	D5S471	129.830	-1.102	0.000	0.000
5	D5S2115	138.640	-0.485	0.000	0.000
5	D5S436	147.490	-0.317	0.000	0.000
5	D5S410	156.470	-0.251	0.000	0.000
5	D5S422	164.190	-0.240	0.000	0.000
5	D5S400	174.800	-0.287	0.000	0.000
5	D5S408	195.490	-1.105	0.000	0.000

Chr: Chromosome; LOD: estimated multipoint LOD score at a particular location;
ALPHA: estimate proportion of linked families (since there is only one informative family in this sample, the proportion will always be 0.000 or 1.000);
HLOD: maximum heterogeneity LOD score

Table 06. Estimated multipoint LOD score at chromosome 06

CHR	MARKER	POSITION (cM)	LOD	ALPHA	HLOD
6	D6S1574	9.180	0.280	1.000	0.280
6	D6S309	14.070	0.280	1.000	0.280
6	D6S470	18.220	0.280	1.000	0.280
6	D6S289	29.930	0.274	1.000	0.274
6	D6S422	35.660	0.280	1.000	0.280
6	D6S276	44.100	-0.035	0.000	0.000
6	D6S1610	53.810	-1.104	0.000	0.000
6	D6S257	79.920	-1.102	0.000	0.000
6	D6S460	89.830	-1.102	0.000	0.000
6	D6S462	99.010	-0.017	0.000	0.000
6	D6S434	109.190	0.287	1.000	0.287
6	D6S287	121.970	0.216	1.000	0.216
6	D6S262	130.000	0.187	1.000	0.187
6	D6S292	139.970	0.229	1.000	0.229
6	D6S308	144.460	0.280	1.000	0.280
6	D6S441	154.100	0.280	1.000	0.280
6	D6S1581	164.780	0.210	1.000	0.210
6	D6S264	179.070	0.133	1.000	0.133
6	D6S446	189.000	0.082	1.000	0.082
6	D6S281	190.140	0.082	1.000	0.082

Chr: Chromosome; LOD: estimated multipoint LOD score at a particular location;
ALPHA: estimate proportion of linked families (since there is only one informative family in this sample, the proportion will always be 0.000 or 1.000);
HLOD: maximum heterogeneity LOD score

Table 07. Estimated multipoint LOD score at chromosome 07

CHR	MARKER	POSITION (cM)	LOD	ALPHA	HLOD
7	D7S531	5.280	-1.105	0.000	0.000
7	D7S517	7.440	-1.105	0.000	0.000
7	D7S513	17.740	-0.923	0.000	0.000
7	D7S507	28.740	-0.929	0.000	0.000
7	D7S493	36.060	-1.105	0.000	0.000
7	D7S516	41.690	-0.987	0.000	0.000
7	D7S484	53.500	-1.105	0.000	0.000
7	D7S510	59.930	0.283	1.000	0.283
7	D7S519	69.030	-1.102	0.000	0.000
7	D7S502	78.650	-1.102	0.000	0.000
7	D7S669	90.420	-1.101	0.000	0.000
7	D7S630	98.440	-1.102	0.000	0.000
7	D7S657	104.860	-0.973	0.000	0.000
7	D7S515	112.320	-1.102	0.000	0.000
7	D7S486	124.080	-0.902	0.000	0.000
7	D7S530	134.550	-1.102	0.000	0.000
7	D7S640	137.830	-1.102	0.000	0.000
7	D7S684	147.220	-1.102	0.000	0.000
7	D7S661	155.100	-1.102	0.000	0.000
7	D7S636	162.330	-1.102	0.000	0.000
7	D7S798	168.980	-0.118	0.000	0.000
7	D7S2465	180.240	0.280	1.000	0.280

Chr: Chromosome; LOD: estimated multipoint LOD score at a particular location;
ALPHA: estimate proportion of linked families (since there is only one informative family in this sample, the proportion will always be 0.000 or 1.000);
HLOD: maximum heterogeneity LOD score

Table 08. Estimated multipoint LOD score at chromosome 08

CHR	MARKER	POSITION (cM)	LOD	ALPHA	HLOD
8	D8S264	0.730	0.287	1.000	0.287
8	D8S277	8.340	-1.105	0.000	0.000
8	D8S550	21.330	-0.874	0.000	0.000
8	D8S549	31.730	-0.718	0.000	0.000
8	D8S258	41.550	-0.745	0.000	0.000
8	D8S1771	50.050	-1.105	0.000	0.000
8	D8S505	60.870	-1.105	0.000	0.000
8	D8S285	71.000	-1.105	0.000	0.000
8	D8S260	79.360	-1.105	0.000	0.000
8	D8S270	103.790	-0.547	0.000	0.000
8	D8S1784	118.150	-1.105	0.000	0.000
8	D8S514	130.000	0.287	1.000	0.287
8	D8S284	143.820	-1.102	0.000	0.000
8	D8S272	154.020	-0.409	0.000	0.000

Chr: Chromosome; LOD: estimated multipoint LOD score at a particular location;
ALPHA: estimate proportion of linked families (since there is only one informative family in this sample, the proportion will always be 0.000 or 1.000);
HLOD: maximum heterogeneity LOD score

Table 09. Estimated multipoint LOD score at chromosome 09

CHR	MARKER	POSITION (cM)	LOD	ALPHA	HLOD
9	D9S288	9.830	0.290	1.000	0.290
9	D9S286	18.060	0.272	1.000	0.272
9	D9S285	29.520	0.282	1.000	0.282
9	D9S157	32.240	0.290	1.000	0.290
9	D9S171	42.730	-1.195	0.000	0.000
9	D9S161	51.810	0.039	1.000	0.039
9	D9S1817	59.340	0.290	1.000	0.290
9	D9S273	65.790	0.288	1.000	0.288
9	D9S175	70.330	0.290	1.000	0.290
9	D9S167	83.410	0.290	1.000	0.290
9	D9S283	94.850	0.290	1.000	0.290
9	D9S287	103.420	0.290	1.000	0.290
9	D9S1690	106.630	0.290	1.000	0.290
9	D9S1677	117.330	0.290	1.000	0.290
9	D9S1776	123.330	0.224	1.000	0.224
9	D9S1682	132.090	-0.056	0.000	0.000
9	D9S290	140.860	-1.179	0.000	0.000
9	D9S164	147.910	-1.195	0.000	0.000
9	D9S1826	159.610	-0.381	0.000	0.000
9	D9S158	161.710	-0.334	0.000	0.000

Chr: Chromosome; LOD: estimated multipoint LOD score at a particular location; ALPHA: estimate proportion of linked families (since there is only one informative family in this sample, the proportion will always be 0.000 or 1.000); HLOD: maximum heterogeneity LOD score

Table 10. Estimated multipoint LOD score at chromosome 10

CHR	MARKER	POSITION (cM)	LOD	ALPHA	HLOD
10	D10S249	2.130	0.287	1.000	0.287
10	D10S591	13.430	0.287	1.000	0.287
10	D10S189	19.000	0.277	1.000	0.277
10	D10S547	29.150	0.198	1.000	0.198
10	D10S1653	40.360	-1.105	0.000	0.000
10	D10S548	45.700	-0.208	0.000	0.000
10	D10S197	52.100	0.280	1.000	0.280
10	D10S208	60.640	0.280	1.000	0.280
10	D10S196	70.230	0.280	1.000	0.280
10	D10S1652	80.770	0.280	1.000	0.280
10	D10S537	91.130	0.280	1.000	0.280
10	D10S1686	105.040	0.280	1.000	0.280
10	D10S185	116.340	0.285	1.000	0.285
10	D10S192	124.270	0.287	1.000	0.287
10	D10S597	128.730	0.115	1.000	0.115
10	D10S1693	137.390	-1.101	0.000	0.000
10	D10S587	147.570	-1.102	0.000	0.000
10	D10S217	157.890	-1.100	0.000	0.000
10	D10S1651	168.770	-1.102	0.000	0.000
10	D10S212	170.940	-0.807	0.000	0.000

Chr: Chromosome; LOD: estimated multipoint LOD score at a particular location;
ALPHA: estimate proportion of linked families (since there is only one informative family in this sample, the proportion will always be 0.000 or 1.000);
HLOD: maximum heterogeneity LOD score

Table 11. Estimated multipoint LOD score at chromosome 11

CHR	MARKER	POSITION (cM)	LOD	ALPHA	HLOD
11	D11S4046	2.790	-1.105	0.000	0.000
11	D11S1338	12.920	-0.884	0.000	0.000
11	D11S902	21.470	-0.933	0.000	0.000
11	D11S904	33.570	-1.102	0.000	0.000
11	D11S935	45.940	-1.102	0.000	0.000
11	D11S905	51.950	-1.102	0.000	0.000
11	D11S4191	60.090	0.022	1.000	0.022
11	D11S987	67.480	0.287	1.000	0.287
11	D11S1314	73.640	0.287	1.000	0.287
11	D11S937	79.980	0.284	1.000	0.284
11	D11S901	85.480	0.287	1.000	0.287
11	D11S4175	91.470	0.287	1.000	0.287
11	D11S898	98.980	-1.102	0.000	0.000
11	D11S908	108.590	-1.102	0.000	0.000
11	D11S925	118.470	-1.102	0.000	0.000
11	D11S4151	127.330	-0.635	0.000	0.000
11	D11S1320	141.910	-0.242	0.000	0.000
11	D11S968	147.770	-0.179	0.000	0.000

Chr: Chromosome; LOD: estimated multipoint LOD score at a particular location;
ALPHA: estimate proportion of linked families (since there is only one informative family in this sample, the proportion will always be 0.000 or 1.000);
HLOD: maximum heterogeneity LOD score

Table 12. Estimated multipoint LOD score at chromosome 12

CHR	MARKER	POSITION (cM)	LOD	ALPHA	HLOD
12	D12S352	0.000	-1.120	0.000	0.000
12	D12S99	12.600	-0.796	0.000	0.000
12	D12S336	19.680	-1.342	0.000	0.000
12	D12S364	30.600	-1.026	0.000	0.000
12	D12S310	36.060	-0.802	0.000	0.000
12	D12S1617	44.020	-0.677	0.000	0.000
12	D12S345	53.090	-1.364	0.000	0.000
12	D12S85	61.340	-1.894	0.000	0.000
12	D12S368	66.030	-1.576	0.000	0.000
12	D12S83	75.170	-1.365	0.000	0.000
12	D12S326	86.400	-1.556	0.000	0.000
12	D12S351	95.560	0.041	1.000	0.041
12	D12S346	104.550	0.202	1.000	0.202
12	D12S78	111.870	0.083	1.000	0.083
12	D12S79	125.310	-0.974	0.000	0.000
12	D12S86	134.540	-0.745	0.000	0.000
12	D12S324	147.170	-1.014	0.000	0.000
12	D12S1659	155.940	-0.987	0.000	0.000
12	D12S1723	164.630	-0.435	0.000	0.000

Chr: Chromosome; LOD: estimated multipoint LOD score at a particular location;
ALPHA: estimate proportion of linked families (since there is only one informative family in this sample, the proportion will always be 0.000 or 1.000);
HLOD: maximum heterogeneity LOD score

Table 13. Estimated multipoint LOD score at chromosome 13

CHR	MARKER	POSITION (cM)	LOD	ALPHA	HLOD
13	D13S175	6.030	-0.146	0.000	0.000
13	D13S217	17.210	-0.799	0.000	0.000
13	D13S171	25.080	-2.282	0.000	0.000
13	D13S218	32.900	-4.190	0.000	0.000
13	D13S263	38.320	-5.178	0.000	0.000
13	D13S153	45.550	-4.383	0.000	0.000
13	D13S156	55.850	-1.526	0.000	0.000
13	D13S170	63.900	-1.517	0.000	0.000
13	D13S265	68.730	-1.608	0.000	0.000
13	D13S159	79.490	-3.447	0.000	0.000
13	D13S158	84.870	-2.164	0.000	0.000
13	D13S173	93.520	-1.080	0.000	0.000
13	D13S1265	98.820	-1.429	0.000	0.000
13	D13S285	110.550	-1.438	0.000	0.000

Chr: Chromosome; LOD: estimated multipoint LOD score at a particular location;
ALPHA: estimate proportion of linked families (since there is only one informative family in this sample, the proportion will always be 0.000 or 1.000);
HLOD: maximum heterogeneity LOD score

Table 14. Estimated multipoint LOD score at chromosome 14

CHR	MARKER	POSITION (cM)	LOD	ALPHA	HLOD
14	D14S261	6.460	-1.097	0.000	0.000
14	D14S283	13.890	-1.102	0.000	0.000
14	D14S275	28.010	-0.674	0.000	0.000
14	D14S70	40.110	0.280	1.000	0.280
14	D14S288	47.510	-1.105	0.000	0.000
14	D14S276	56.360	-1.105	0.000	0.000
14	D14S63	69.180	-1.105	0.000	0.000
14	D14S258	76.280	-1.105	0.000	0.000
14	D14S74	87.360	-0.557	0.000	0.000
14	D14S68	95.890	-0.479	0.000	0.000
14	D14S280	105.000	-0.516	0.000	0.000
14	D14S65	117.300	-0.924	0.000	0.000
14	D14S985	126.610	-1.103	0.000	0.000
14	D14S292	134.300	-0.491	0.000	0.000

Chr: Chromosome; LOD: estimated multipoint LOD score at a particular location;
ALPHA: estimate proportion of linked families (since there is only one informative family in this sample, the proportion will always be 0.000 or 1.000);
HLOD: maximum heterogeneity LOD score

Table 15. Estimated multipoint LOD score at chromosome 15

CHR	MARKER	POSITION (cM)	LOD	ALPHA	HLOD
15	D15S128	6.110	0.281	1.000	0.281
15	D15S1002	14.580	-0.325	0.000	0.000
15	D15S165	20.240	-1.105	0.000	0.000
15	D15S1007	25.860	-1.105	0.000	0.000
15	D15S118	32.580	-1.082	0.000	0.000
15	D15S1012	35.950	-1.083	0.000	0.000
15	D15S994	40.250	-1.102	0.000	0.000
15	D15S161	45.620	-0.996	0.000	0.000
15	D15S978	45.620	-0.996	0.000	0.000
15	D15S117	51.210	-1.102	0.000	0.000
15	D15S153	62.400	0.287	1.000	0.287
15	D15S131	71.280	0.287	1.000	0.287
15	D15S205	78.920	-1.102	0.000	0.000
15	D15S127	86.810	-1.102	0.000	0.000
15	D15S130	100.59	-1.105	0.000	0.000
15	D15S120	112.58	-1.105	0.000	0.000

Chr: Chromosome; LOD: estimated multipoint LOD score at a particular location;
ALPHA: estimate proportion of linked families (since there is only one informative family in this sample, the proportion will always be 0.000 or 1.000);
HLOD: maximum heterogeneity LOD score

Table 16. Estimated multipoint LOD score at chromosome 16

CHR	MARKER	POSITION (cM)	LOD	ALPHA	HLOD
16	D16S423	10.360	-1.102	0.000	0.000
16	D16S404	18.070	-1.130	0.000	0.000
16	D16S3075	23.280	-1.095	0.000	0.000
16	D16S3103	32.070	-1.105	0.000	0.000
16	D16S3046	40.650	-0.990	0.000	0.000
16	D16S3068	48.530	-0.721	0.000	0.000
16	D16S3136	62.110	-1.103	0.000	0.000
16	D16S415	67.620	-0.947	0.000	0.000
16	D16S503	83.550	-0.688	0.000	0.000
16	D16S515	92.100	0.033	1.000	0.033
16	D16S516	100.390	0.287	1.000	0.287
16	D16S3091	111.120	0.287	1.000	0.287
16	D16S520	125.820	-1.102	0.000	0.000

Chr: Chromosome; LOD: estimated multipoint LOD score at a particular location;
ALPHA: estimate proportion of linked families (since there is only one informative family in this sample, the proportion will always be 0.000 or 1.000);
HLOD: maximum heterogeneity LOD score

Table 17. Estimated multipoint LOD score at chromosome 17

CHR	MARKER	POSITION (cM)	LOD	ALPHA	HLOD
17	D17S849	0.630	-1.673	0.000	0.000
17	D17S831	6.600	-2.161	0.000	0.000
17	D17S938	14.690	-0.220	0.000	0.000
17	D17S1852	22.240	-1.761	0.000	0.000
17	D17S799	31.960	-2.114	0.000	0.000
17	D17S921	36.140	-2.341	0.000	0.000
17	D17S1358	37.440	-3.235	0.000	0.000
17	D17S1357	39.000	-3.156	0.000	0.000
17	D17S122	39.260	-3.121	0.000	0.000
17	D17S1857	43.010	-2.975	0.000	0.000
17	D17S798	53.410	-2.245	0.000	0.000
17	D17S1868	64.160	-1.875	0.000	0.000
17	D17S787	74.990	-1.849	0.000	0.000
17	D17S944	82.560	-1.943	0.000	0.000
17	D17S949	93.270	-3.126	0.000	0.000
17	D17S785	103.560	-0.862	0.000	0.000
17	D17S784	116.860	-0.922	0.000	0.000
17	D17S928	126.460	-1.885	0.000	0.000

Chr: Chromosome; LOD: estimated multipoint LOD score at a particular location;
ALPHA: estimate proportion of linked families (since there is only one informative family in this sample, the proportion will always be 0.000 or 1.000);
HLOD: maximum heterogeneity LOD score

Table 18. Estimated multipoint LOD score at chromosome 18

CHR	MARKER	POSITION (cM)	LOD	ALPHA	HLOD
18	D18S59	0.000	0.287	1.000	0.287
18	D18S63	8.300	0.162	1.000	0.162
18	D18S452	18.700	-0.068	0.000	0.000
18	D18S464	31.170	-0.862	0.000	0.000
18	D18S53	41.240	-0.872	0.000	0.000
18	D18S478	52.860	-1.102	0.000	0.000
18	D18S1102	62.840	-0.198	0.000	0.000
18	D18S474	71.320	-1.103	0.000	0.000
18	D18S64	84.800	-0.452	0.000	0.000
18	D18S68	96.480	-0.327	0.000	0.000
18	D18S61	105.030	-0.295	0.000	0.000
18	D18S1161	114.260	-0.297	0.000	0.000
18	D18S462	120.050	-0.290	0.000	0.000
18	D18S70	126.000	-0.296	0.000	0.000

Chr: Chromosome; LOD: estimated multipoint LOD score at a particular location;
ALPHA: estimate proportion of linked families (since there is only one informative family in this sample, the proportion will always be 0.000 or 1.000);
HLOD: maximum heterogeneity LOD score

Table 19. Estimated multipoint LOD score at chromosome 19

CHR	MARKER	POSITION (cM)	LOD	ALPHA	HLOD
19	D19S209	10.970	-3.380	0.000	0.000
19	D19S216	20.010	-3.967	0.000	0.000
19	D19S884	26.370	-2.831	0.000	0.000
19	D19S221	36.220	-2.518	0.000	0.000
19	D19S226	42.280	-2.567	1.000	0.009
19	D19S414	54.010	-3.618	0.000	0.000
19	D19S220	62.030	-2.388	0.000	0.000
19	D19S420	66.300	-1.197	0.000	0.000
19	D19S902	72.720	-2.391	0.000	0.000
19	D19S571	84.080	-2.408	0.000	0.000
19	D19S418	92.560	-1.923	0.000	0.000
19	D19S210	100.010	-1.880	0.000	0.000

Chr: Chromosome; LOD: estimated multipoint LOD score at a particular location;
ALPHA: estimate proportion of linked families (since there is only one informative family in this sample, the proportion will always be 0.000 or 1.000);
HLOD: maximum heterogeneity LOD score

Table 20. Estimated multipoint LOD score at chromosome 20

CHR	MARKER	POSITION (cM)	LOD	ALPHA	HLOD
20	D20S117	2.830	-3.350	0.000	0.000
20	D20S889	11.200	0.865	1.000	0.865
20	D20S115	21.150	-1.686	0.000	0.000
20	D20S186	32.300	-1.663	0.000	0.000
20	D20S112	39.250	0.009	1.000	0.009
20	D20S195	50.810	-1.815	0.000	0.000
20	D20S107	55.740	-2.577	0.000	0.000
20	D20S119	61.770	-3.271	0.000	0.000
20	D20S178	66.160	-1.943	0.000	0.000
20	D20S196	75.010	-1.423	0.000	0.000
20	D20S100	84.780	-0.333	0.000	0.000
20	D20S171	95.700	-0.130	0.000	0.000
20	D20S173	98.090	-0.110	0.000	0.000

Chr: Chromosome; LOD: estimated multipoint LOD score at a particular location;
ALPHA: estimate proportion of linked families (since there is only one informative family in this sample, the proportion will always be 0.000 or 1.000);
HLOD: maximum heterogeneity LOD score

Table 21. Estimated multipoint LOD score at chromosome 21

CHR	MARKER	POSITION (cM)	LOD	ALPHA	HLOD
21	D21S1256	9.720	-2.483	0.000	0.000
21	D21S1914	19.390	-3.972	0.000	0.000
21	D21S263	27.400	-3.942	0.000	0.000
21	D21S1252	35.450	-3.508	0.000	0.000
21	D21S266	45.870	-4.876	0.000	0.000

Chr: Chromosome; LOD: estimated multipoint LOD score at a particular location;
ALPHA: estimate proportion of linked families (since there is only one informative family in this sample, the proportion will always be 0.000 or 1.000);
HLOD: maximum heterogeneity LOD score

Table 22. Estimated multipoint LOD score at chromosome 22

CHR	MARKER	POSITION (cM)	LOD	ALPHA	HLOD
22	D22S420	4.060	-1.617	0.000	0.000
22	D22S539	14.440	-2.087	0.000	0.000
22	D22S315	21.470	-3.586	0.000	0.000
22	D22S280	31.300	-2.249	0.000	0.000
22	D22S283	38.620	-1.387	0.000	0.000
22	D22S423	46.420	-1.033	0.000	0.000
22	D22S274	51.540	-1.340	0.000	0.000

Chr: Chromosome; LOD: estimated multipoint LOD score at a particular location;
ALPHA: estimate proportion of linked families (since there is only one informative family in this sample, the proportion will always be 0.000 or 1.000);
HLOD: maximum heterogeneity LOD score
