# Supplementary material for: Genetic aspects of adolescent idiopathic scoliosis in a family with multiple affected members: a research article
Source: Scoliosis. 2010 Apr 7;5:7. doi: 10.1186/1748-7161-5-7 (PMC2873229; doi:10.1186/1748-7161-5-7)
Supplement: Additional file 2 — Graphics presenting the parametric analysis for dominant model at chromosomes 1 to 22. [file 1748-7161-5-7-S2.PDF]

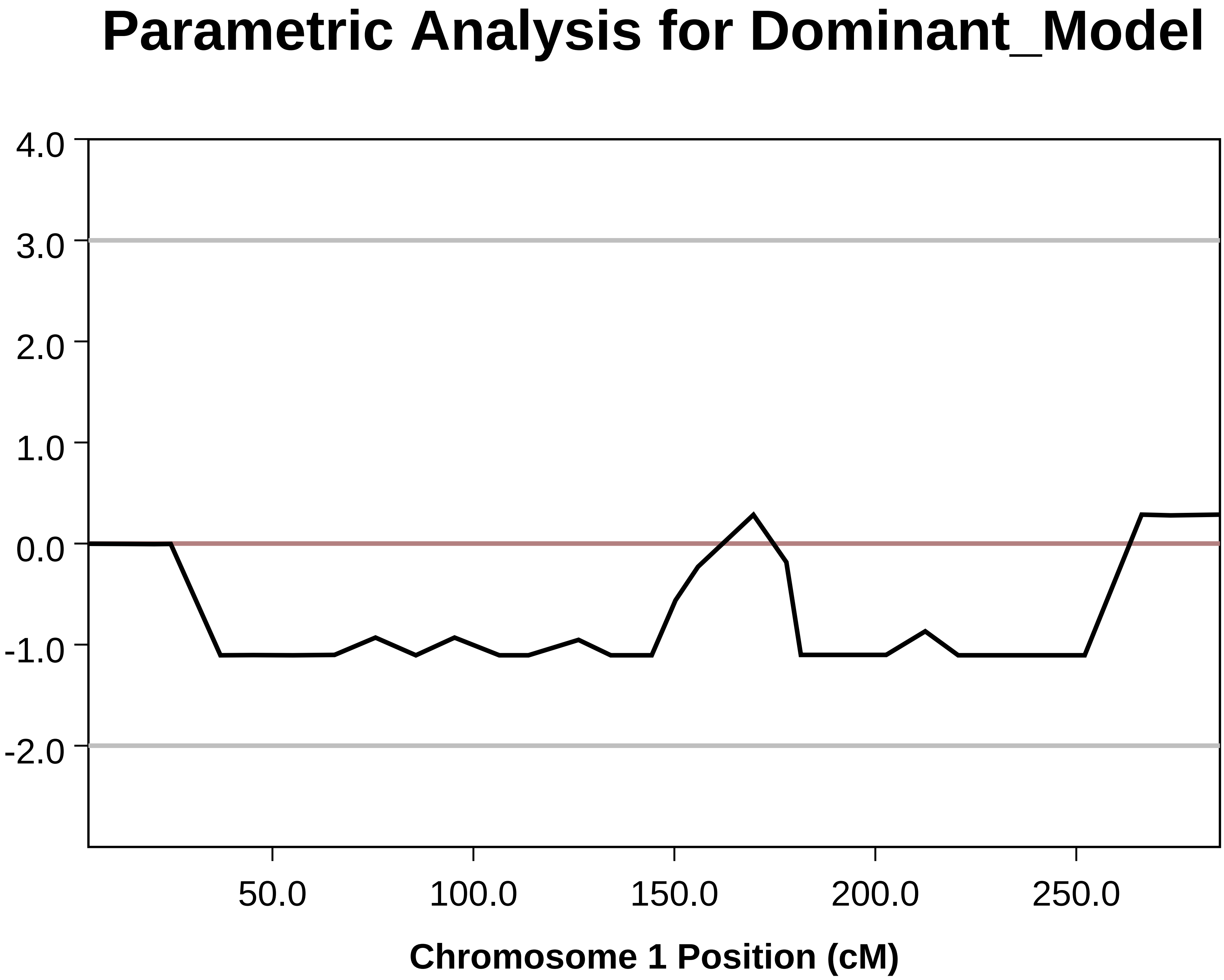

Parametric Analysis for Dominant_Model
4.0
3.0
2.0
1.0
0.0
-1.0
-2.0
50.0
100.0
150.0
200.0
250.0
Chromosome 1 Position (cM)

Parametric Analysis for Dominant_Model

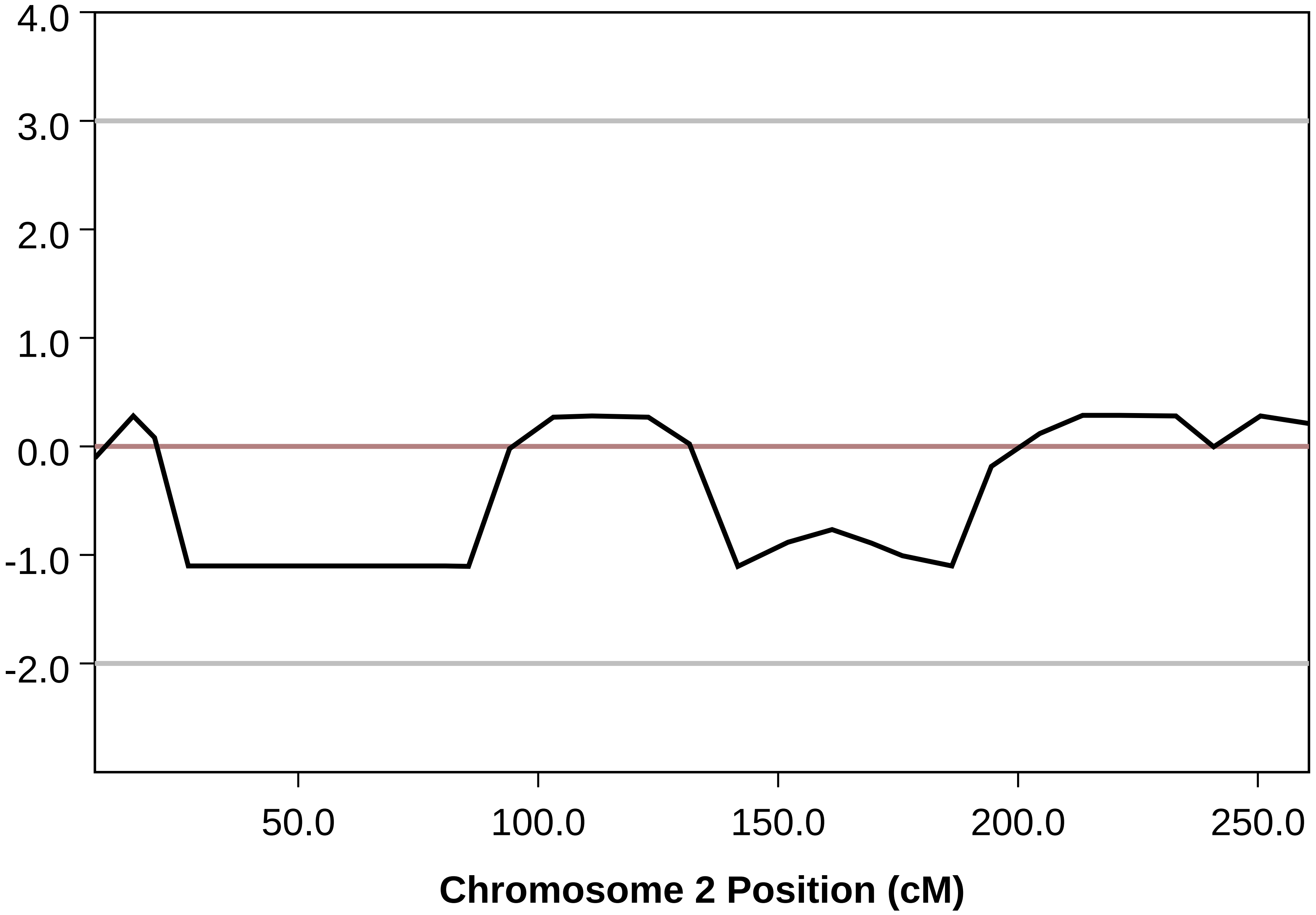

Parametric Analysis for Dominant_Model

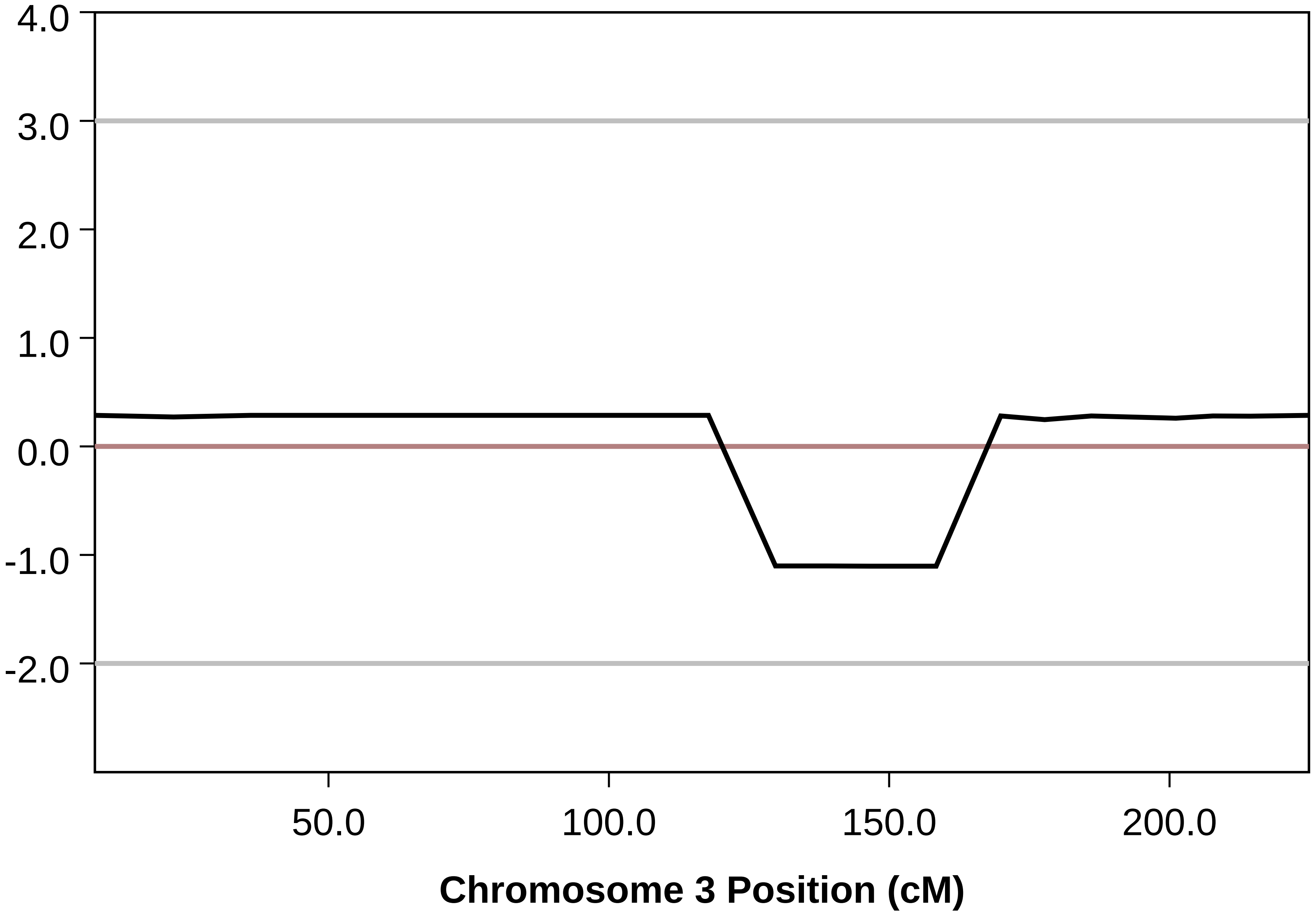

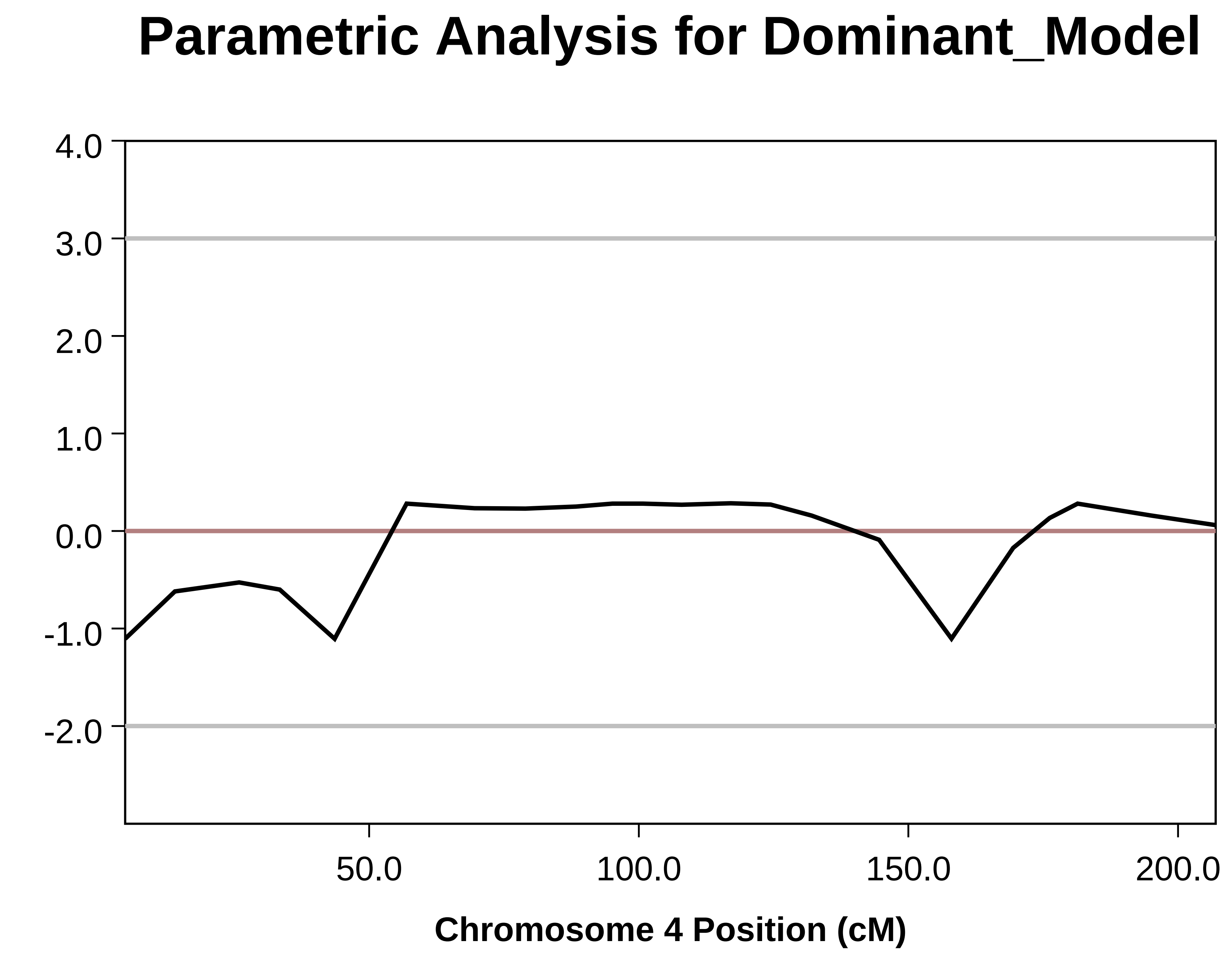

Parametric Analysis for Dominant_Model
4.0
3.0
2.0
1.0
0.0
-1.0
-2.0
50.0
100.0
150.0
200.0
Chromosome 4 Position (cM)

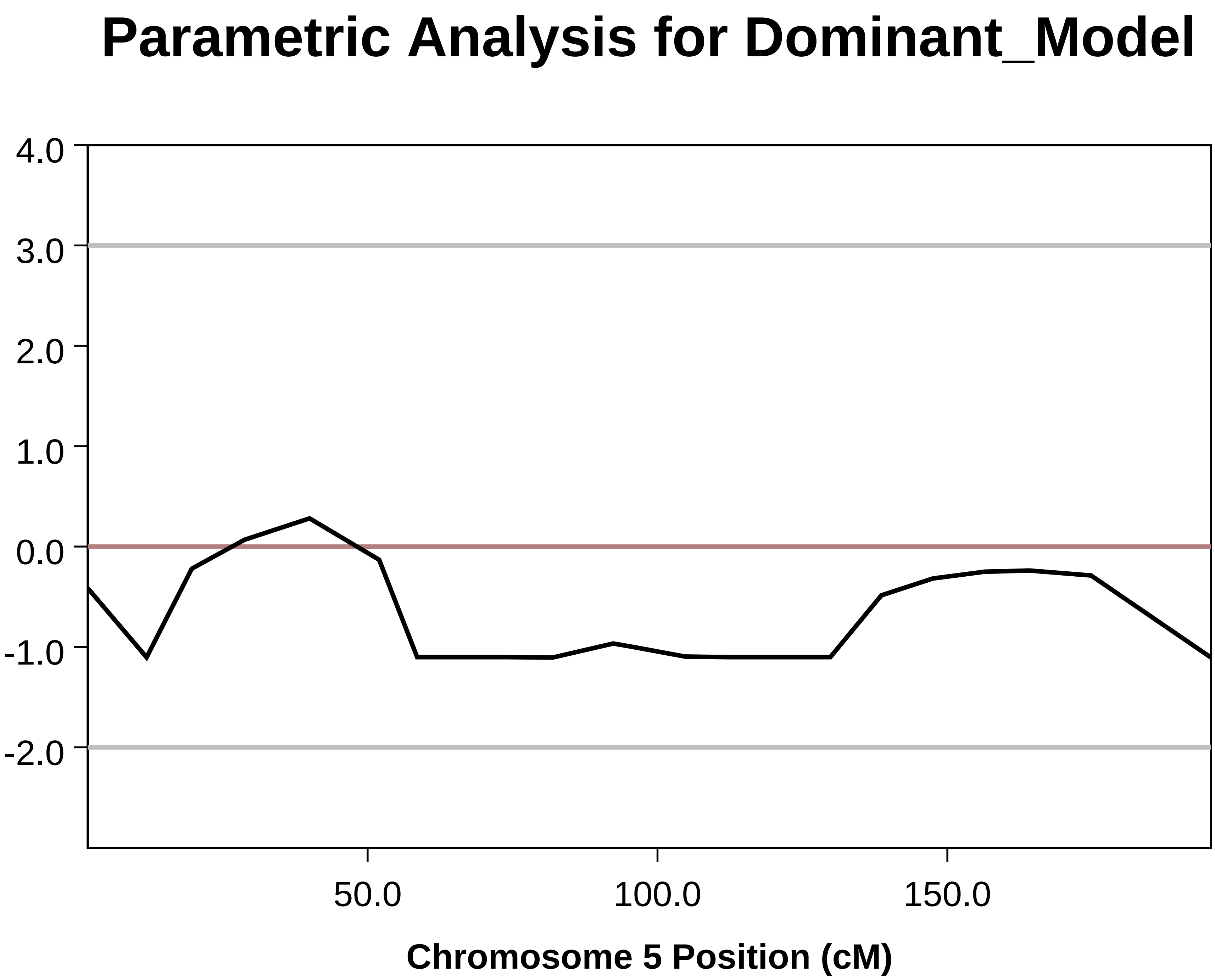
Parametric Analysis for Dominant_Model
4.0
3.0
2.0
1.0
0.0
-1.0
-2.0
50.0
100.0
150.0
Chromosome 5 Position (cM)

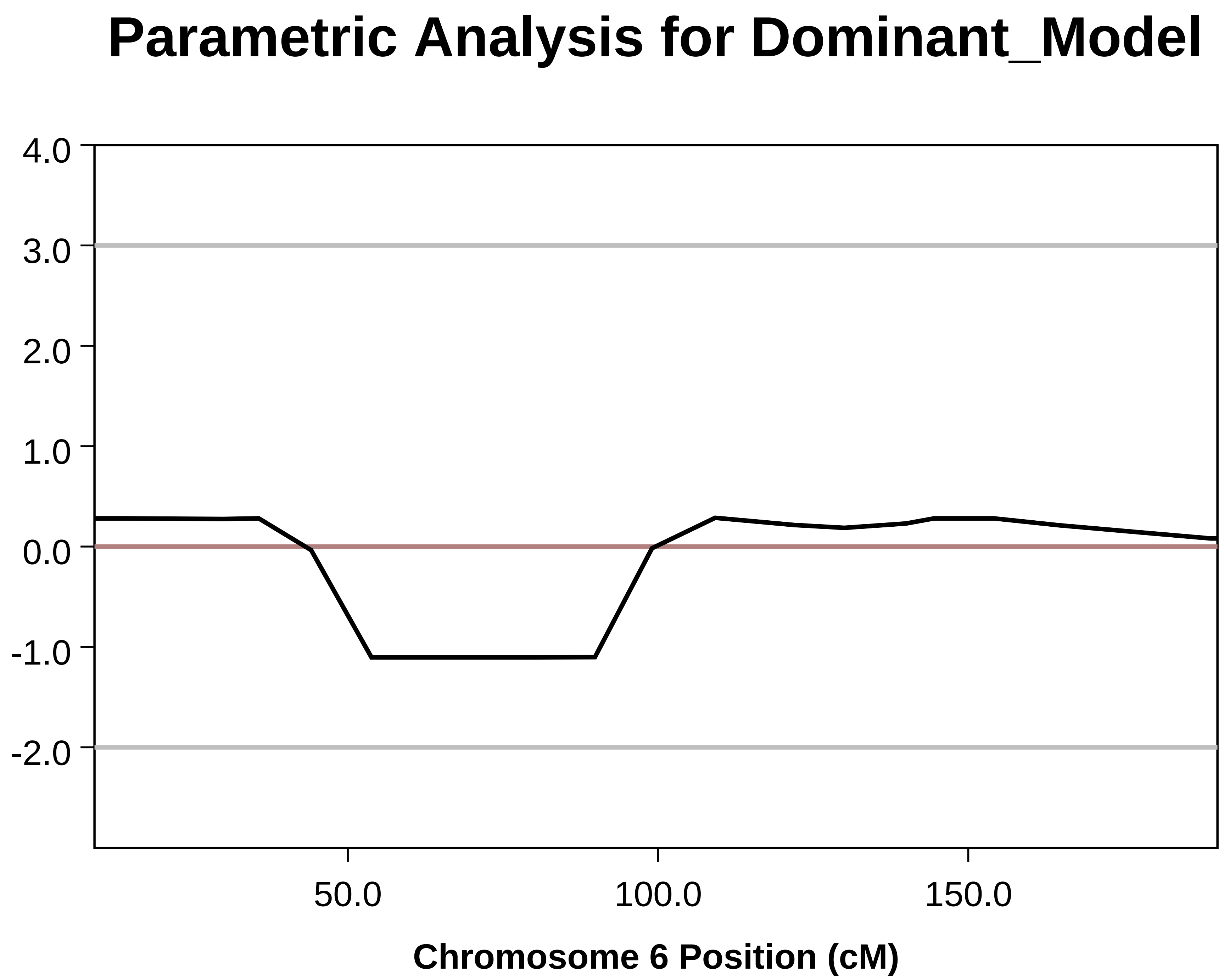
Parametric Analysis for Dominant_Model
4.0
3.0
2.0
1.0
0.0
-1.0
-2.0
50.0
100.0
150.0
Chromosome 6 Position (cM)

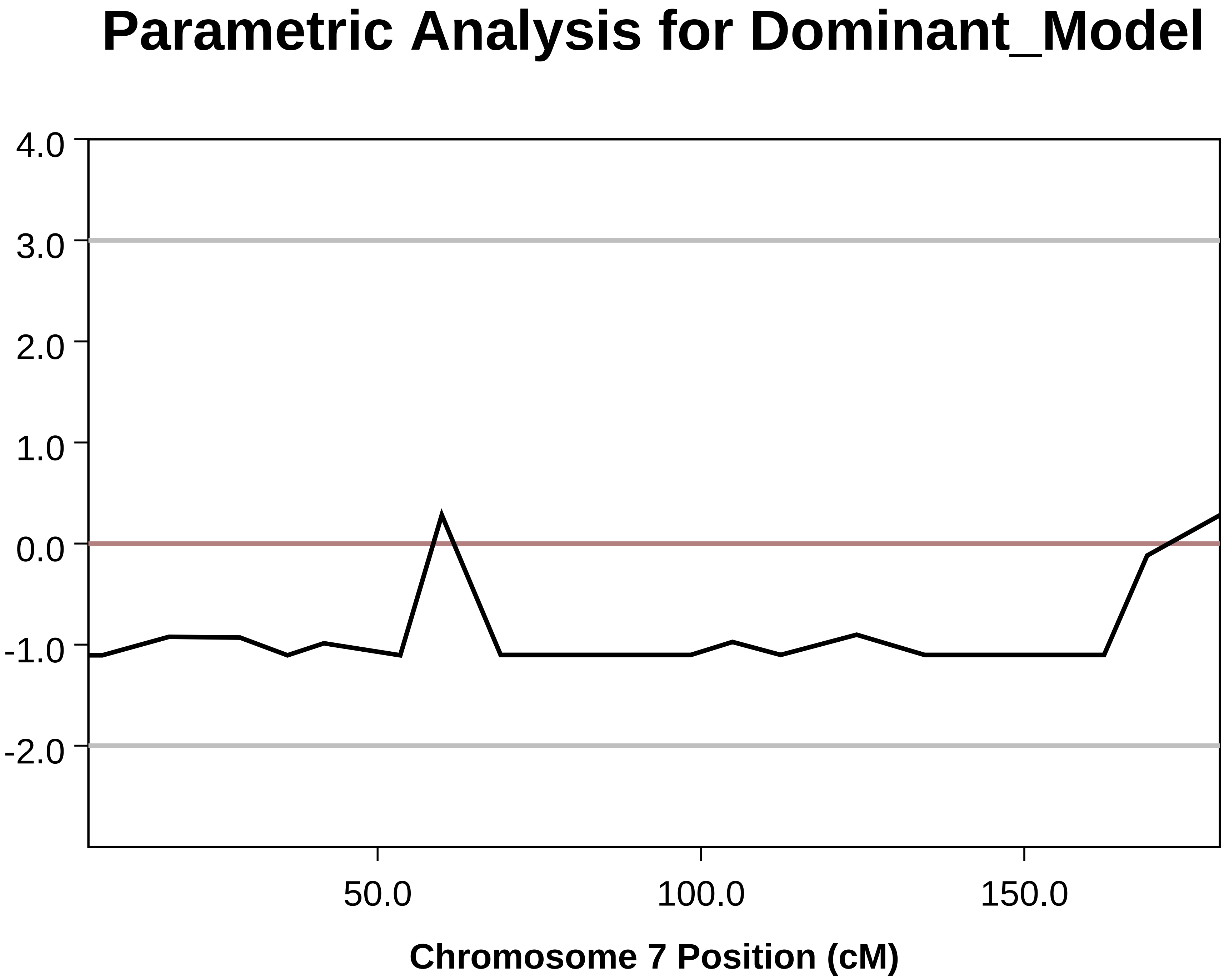

Parametric Analysis for Dominant_Model
4.0
3.0
2.0
1.0
0.0
-1.0
-2.0
50.0
100.0
150.0
Chromosome 7 Position (cM)

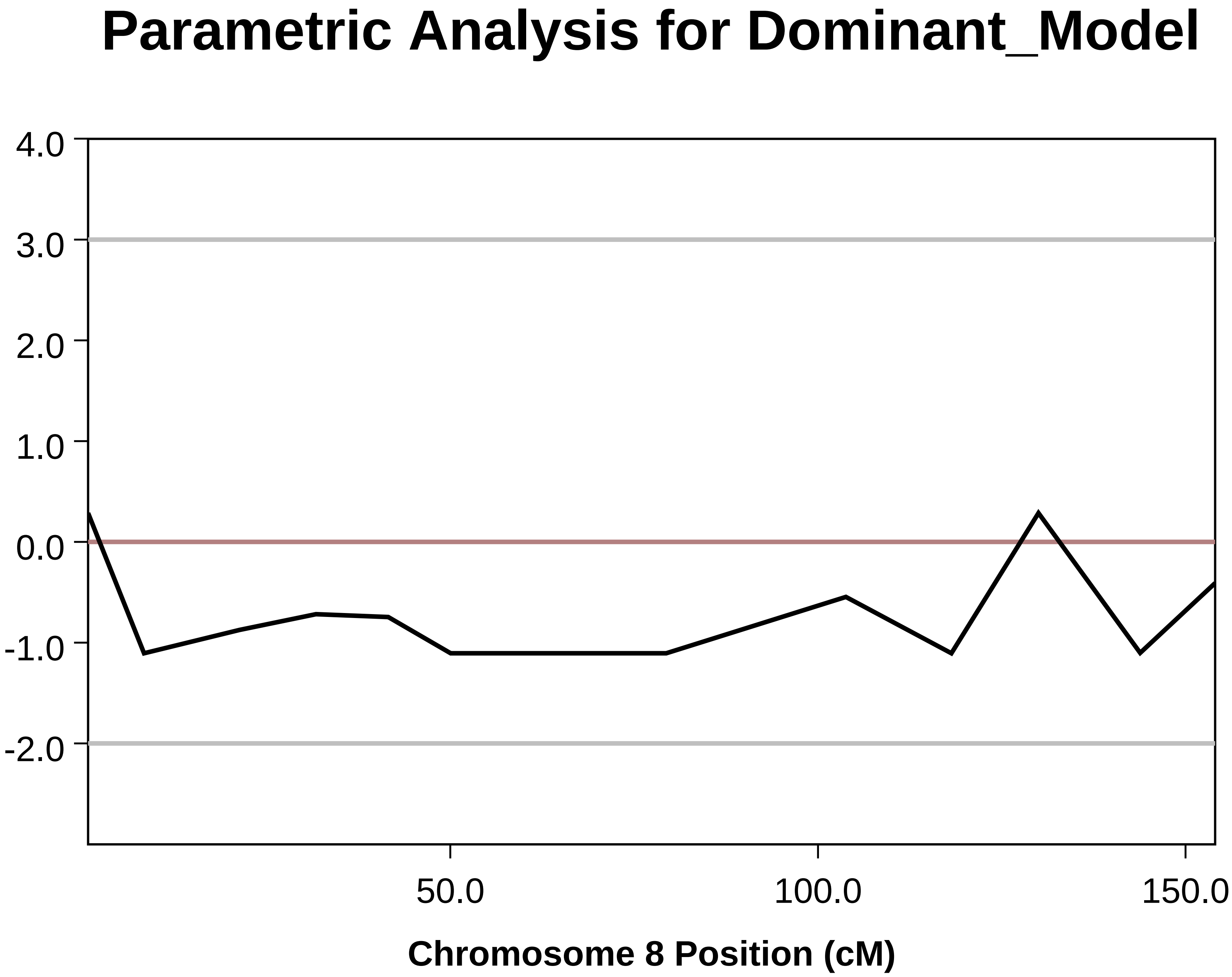
Parametric Analysis for Dominant_Model
4.0
3.0
2.0
1.0
0.0
-1.0
-2.0
50.0
100.0
150.0
Chromosome 8 Position (cM)

Parametric Analysis for Dominant_Model

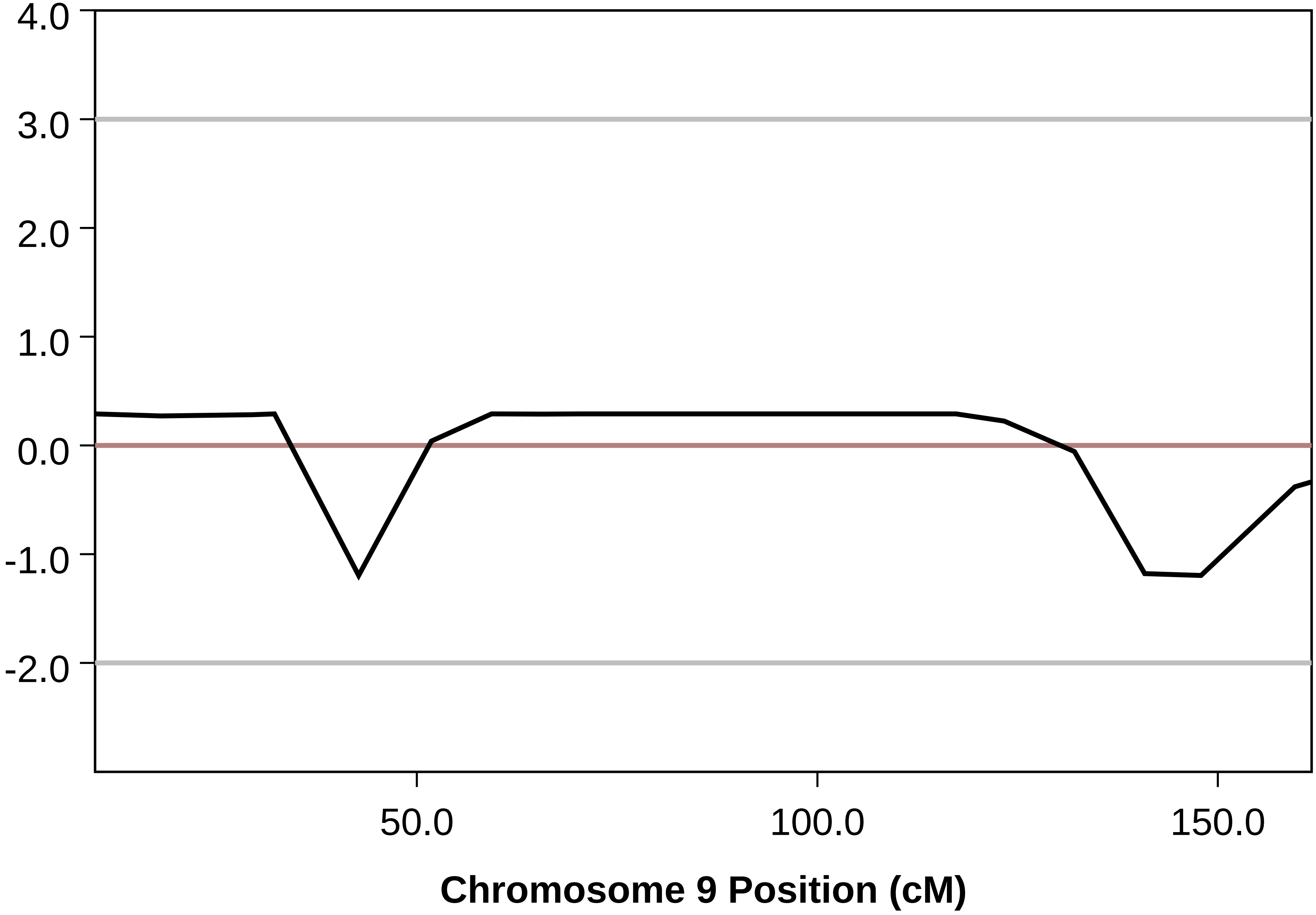

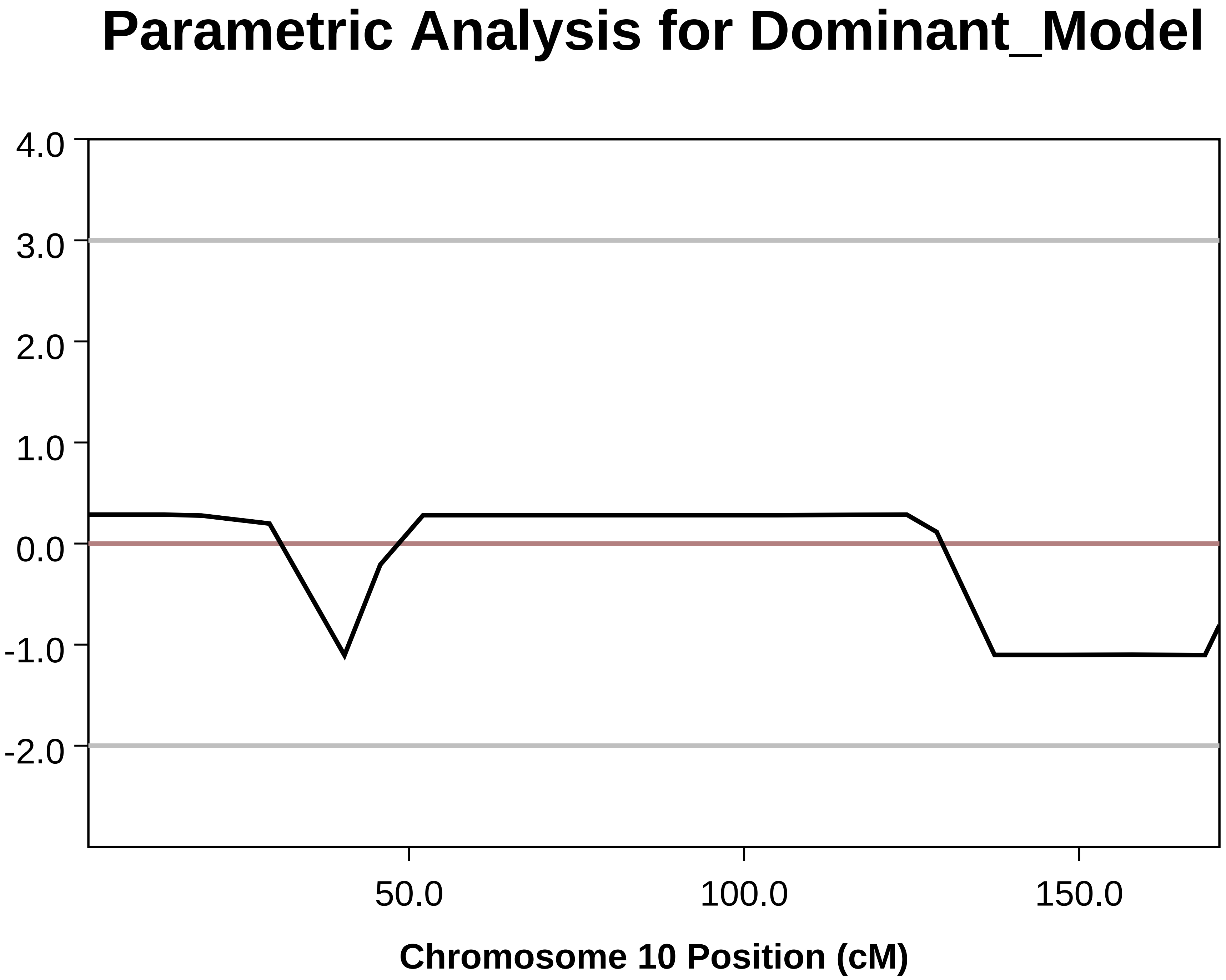

Parametric Analysis for Dominant_Model
4.0
3.0
2.0
1.0
0.0
-1.0
-2.0
50.0
100.0
150.0
Chromosome 10 Position (cM)

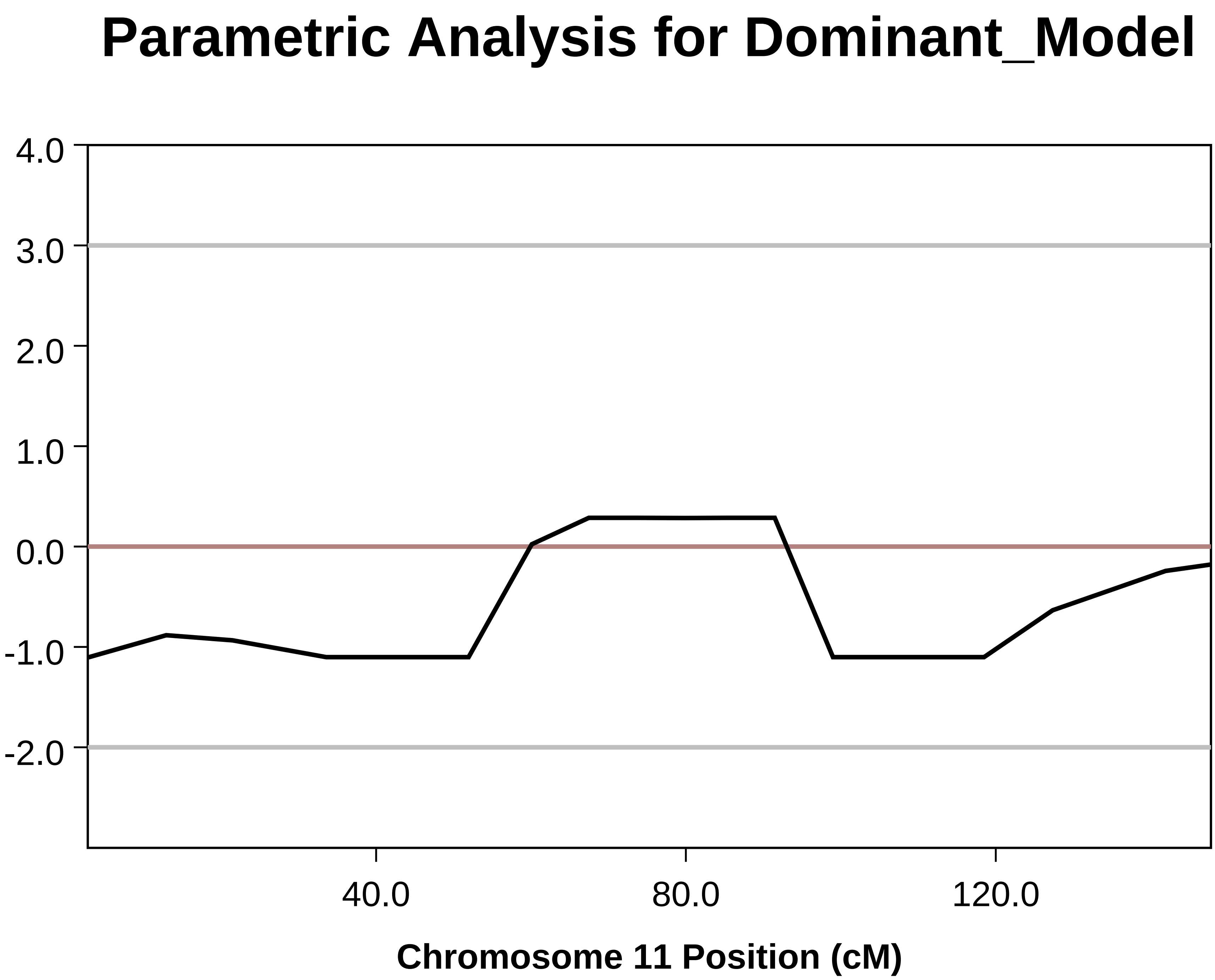

Parametric Analysis for Dominant_Model
4.0
3.0
2.0
1.0
0.0
-1.0
-2.0
40.0
80.0
120.0
Chromosome 11 Position (cM)

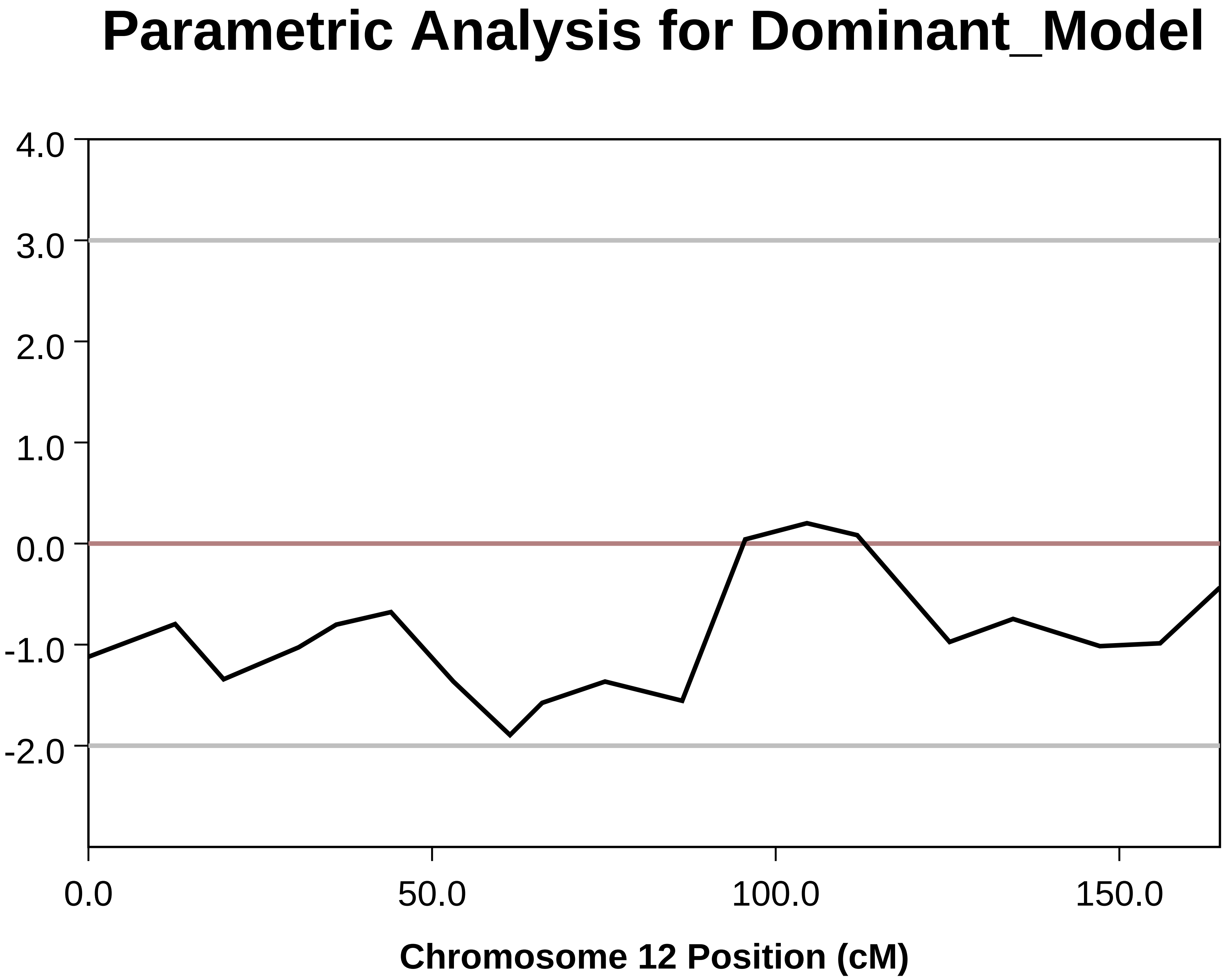
Parametric Analysis for Dominant_Model
4.0
3.0
2.0
1.0
0.0
-1.0
-2.0
0.0
50.0
100.0
150.0
Chromosome 12 Position (cM)

Parametric Analysis for Dominant_Model

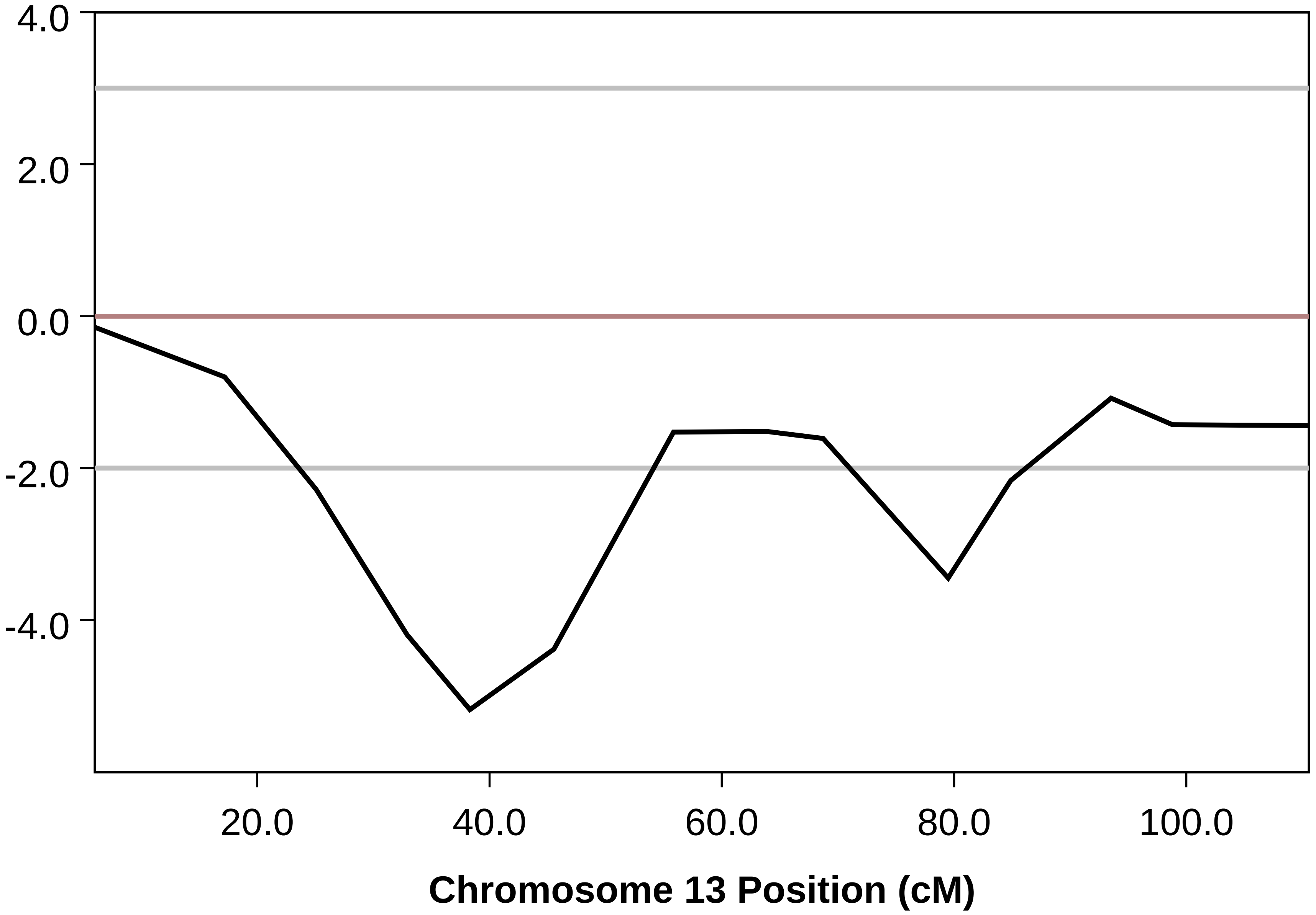

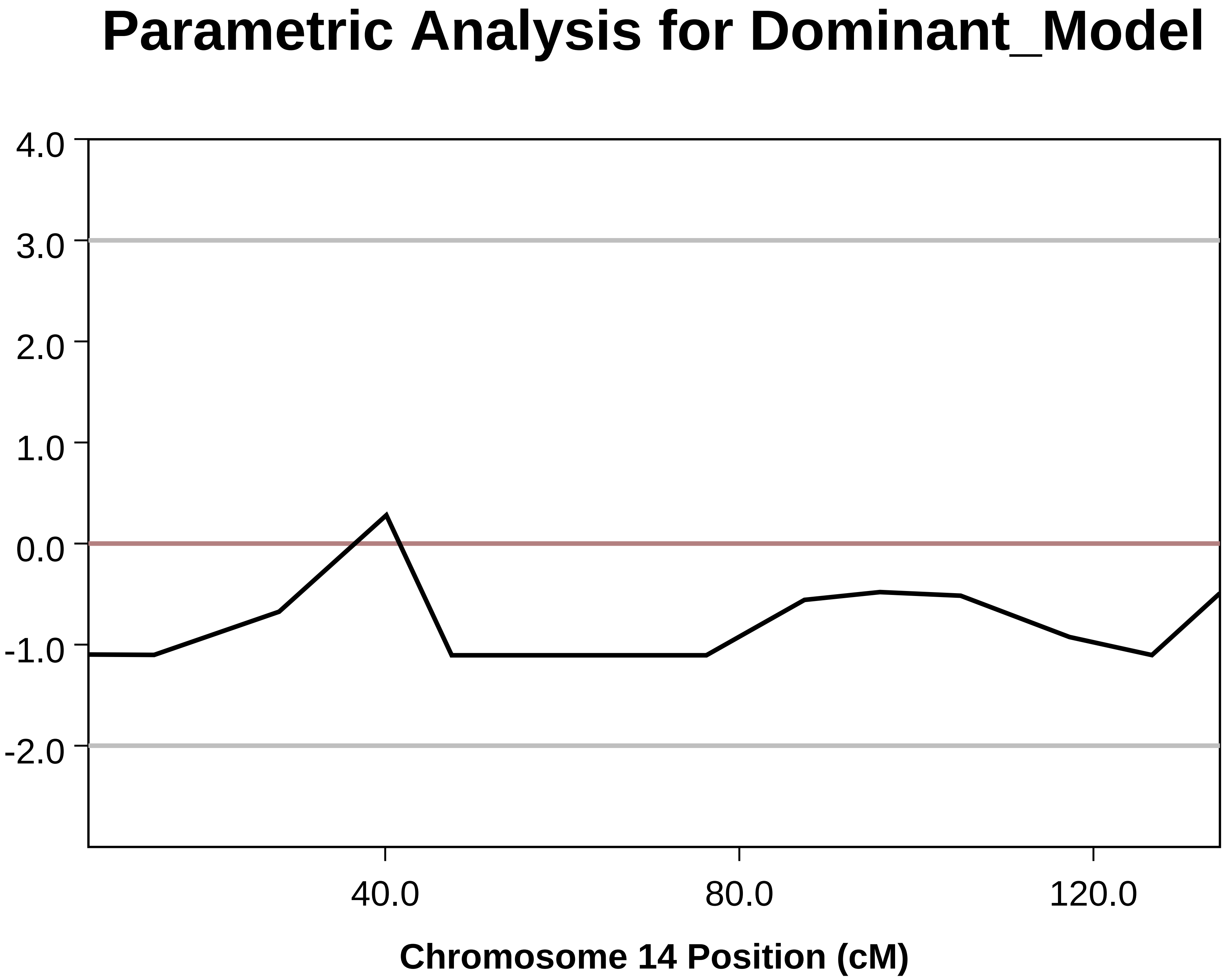

Parametric Analysis for Dominant_Model
4.0
3.0
2.0
1.0
0.0
-1.0
-2.0
40.0
80.0
120.0
Chromosome 14 Position (cM)

Parametric Analysis for Dominant_Model

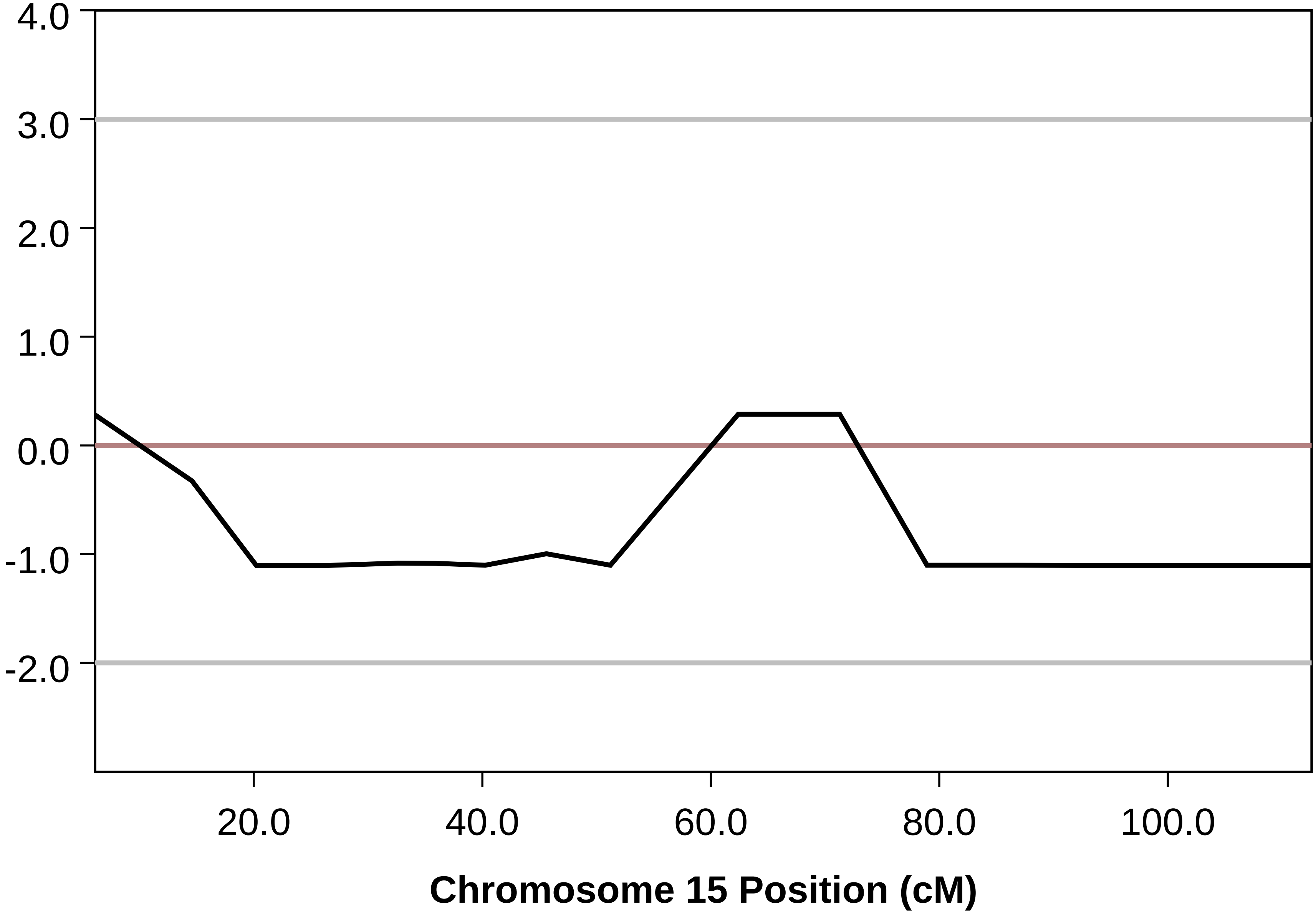

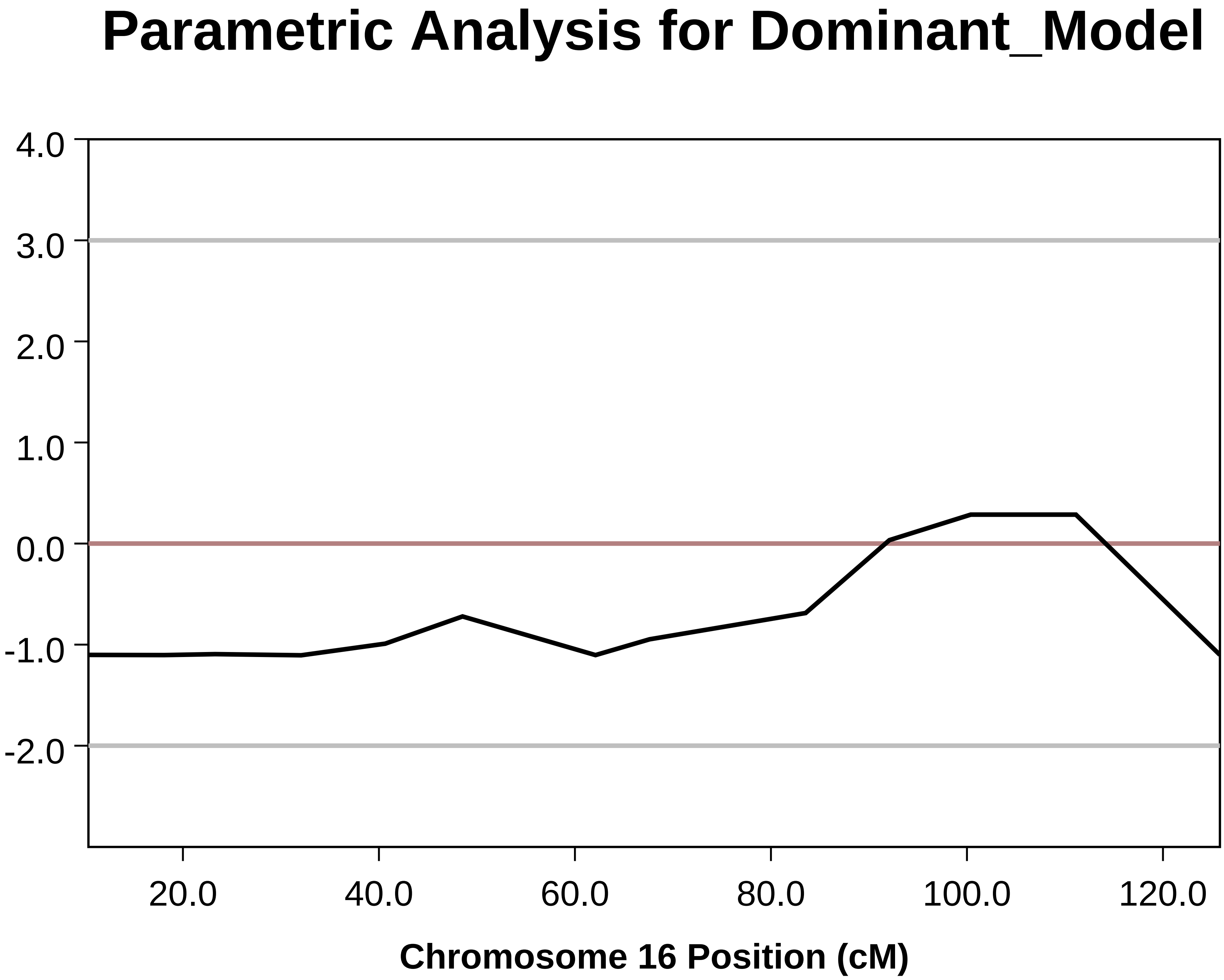
Parametric Analysis for Dominant_Model
4.0
3.0
2.0
1.0
0.0
-1.0
-2.0
20.0
40.0
60.0
80.0
100.0
120.0
Chromosome 16 Position (cM)

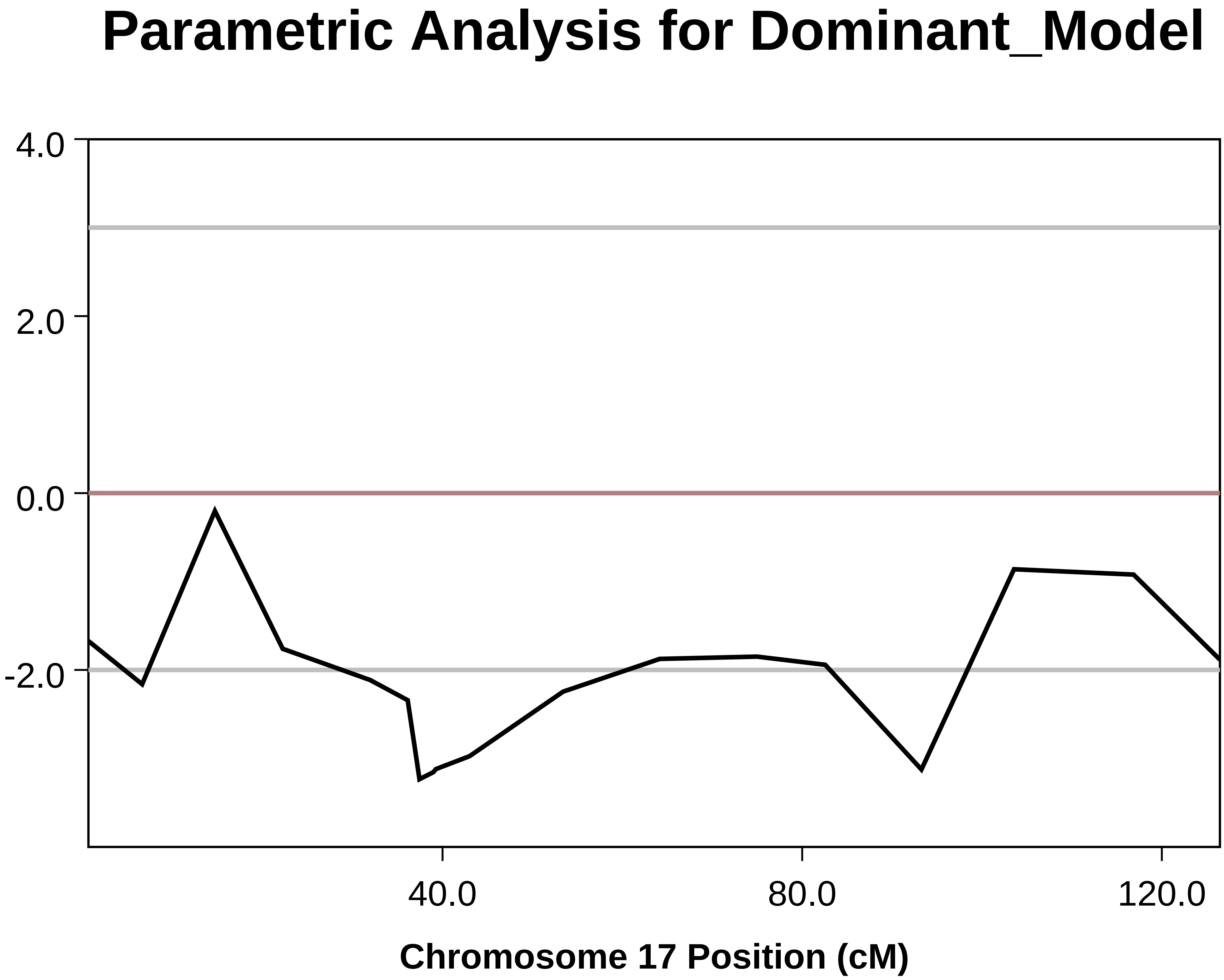
Parametric Analysis for Dominant_Model
4.0
2.0
0.0
-2.0
40.0
80.0
120.0
Chromosome 17 Position (cM)

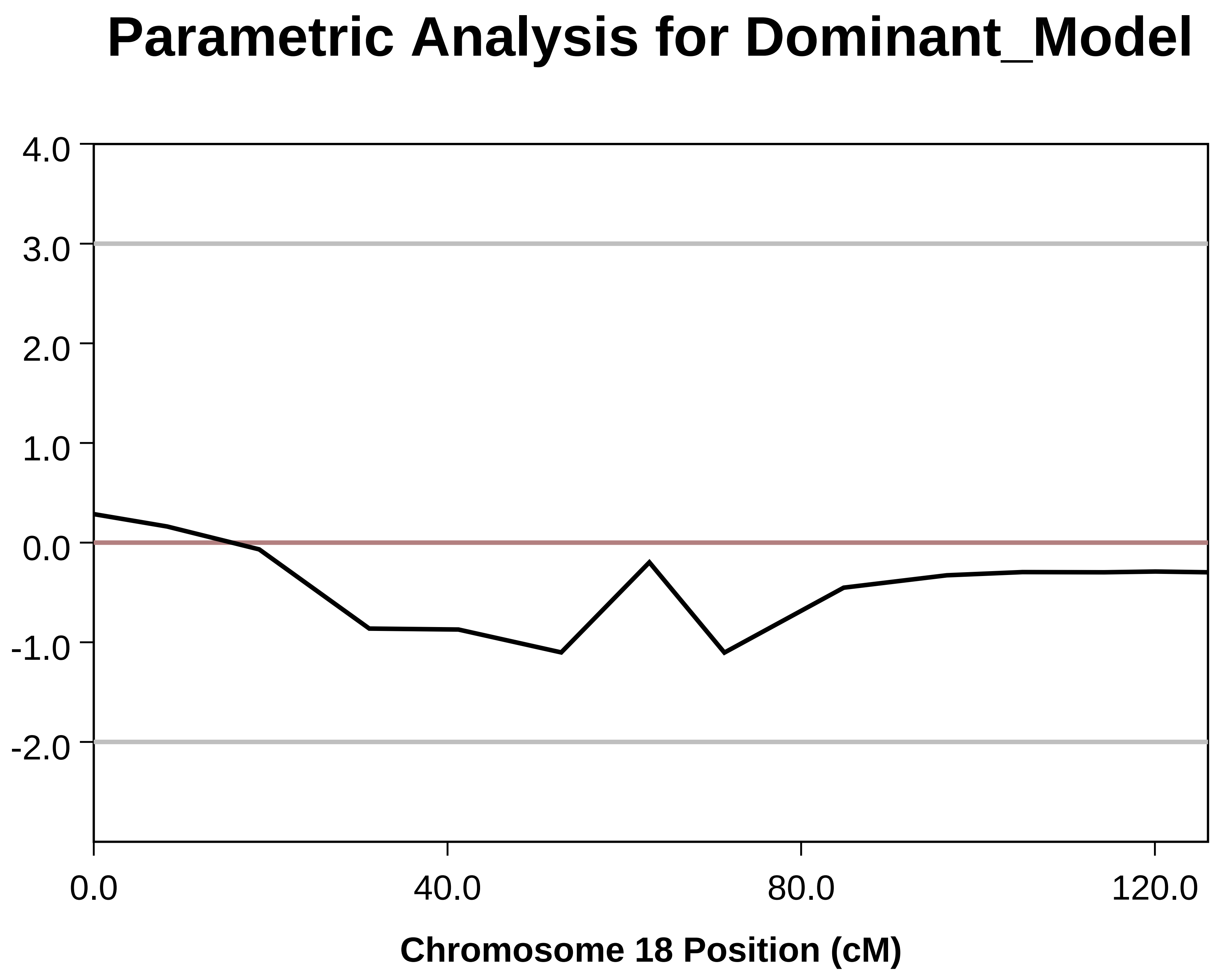

Parametric Analysis for Dominant_Model
4.0
3.0
2.0
1.0
0.0
-1.0
-2.0
0.0
40.0
80.0
120.0
Chromosome 18 Position (cM)

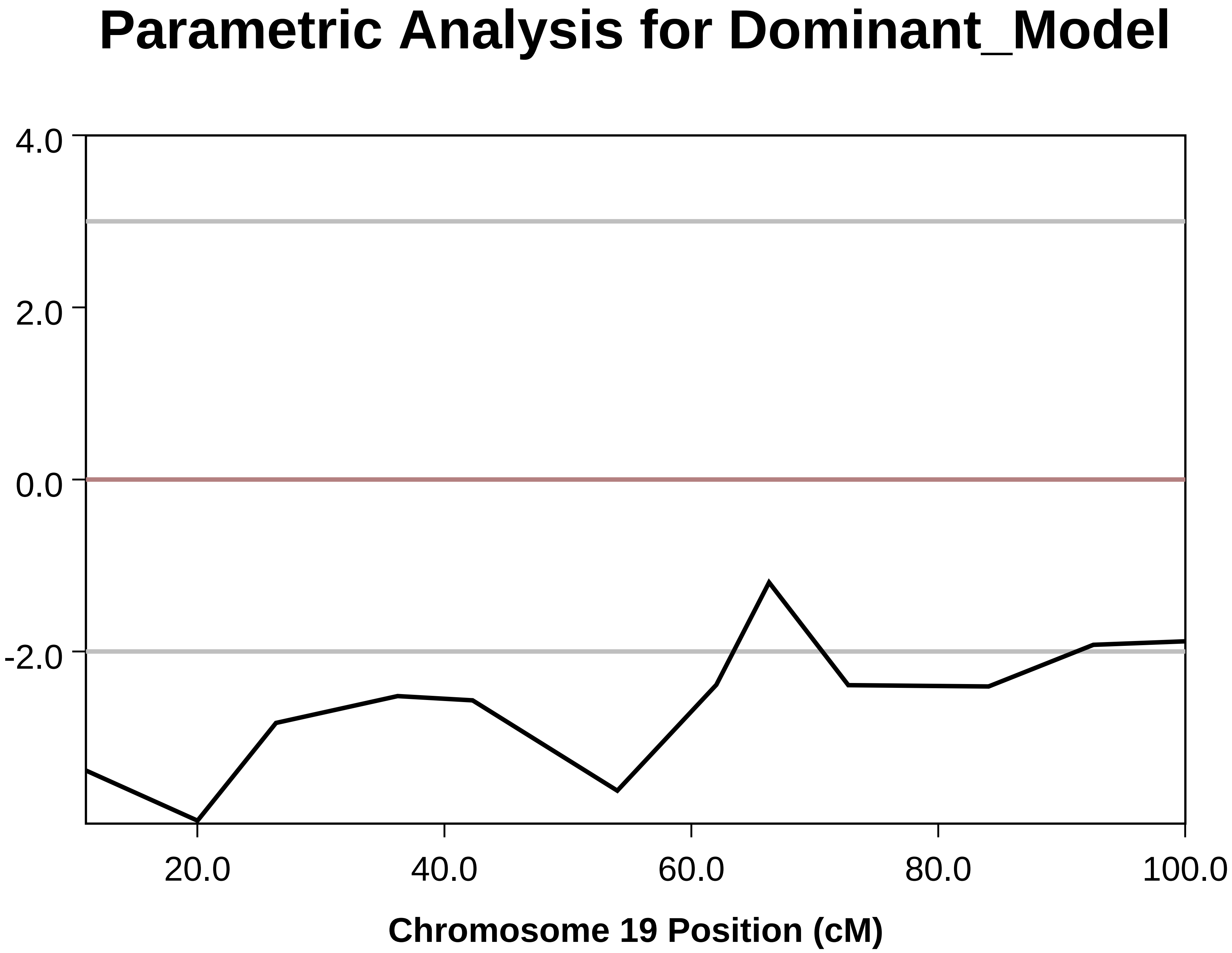
Parametric Analysis for Dominant_Model
4.0
2.0
0.0
-2.0
20.0
40.0
60.0
80.0
100.0
Chromosome 19 Position (cM)

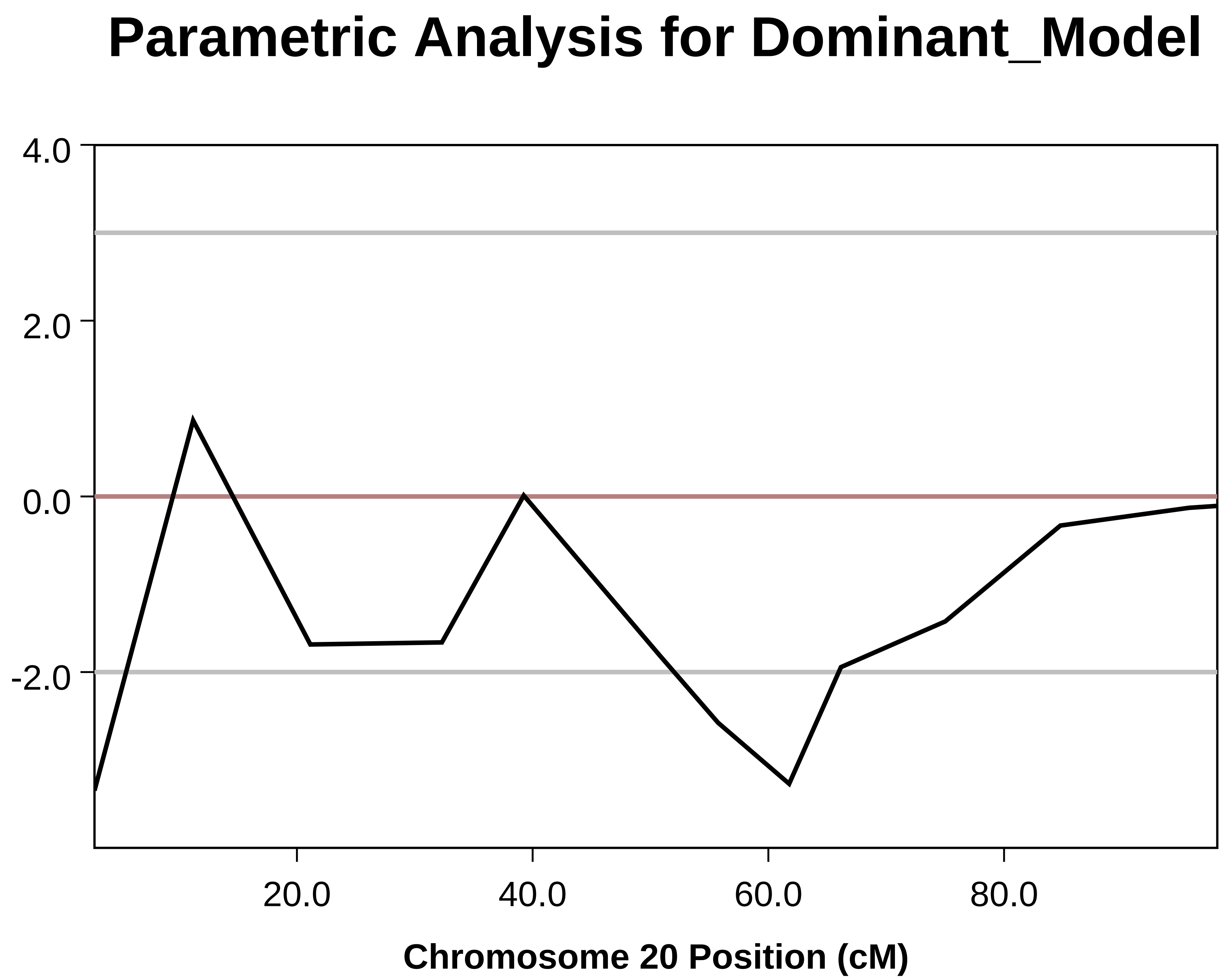
Parametric Analysis for Dominant_Model
4.0
2.0
0.0
-2.0
20.0
40.0
60.0
80.0
Chromosome 20 Position (cM)

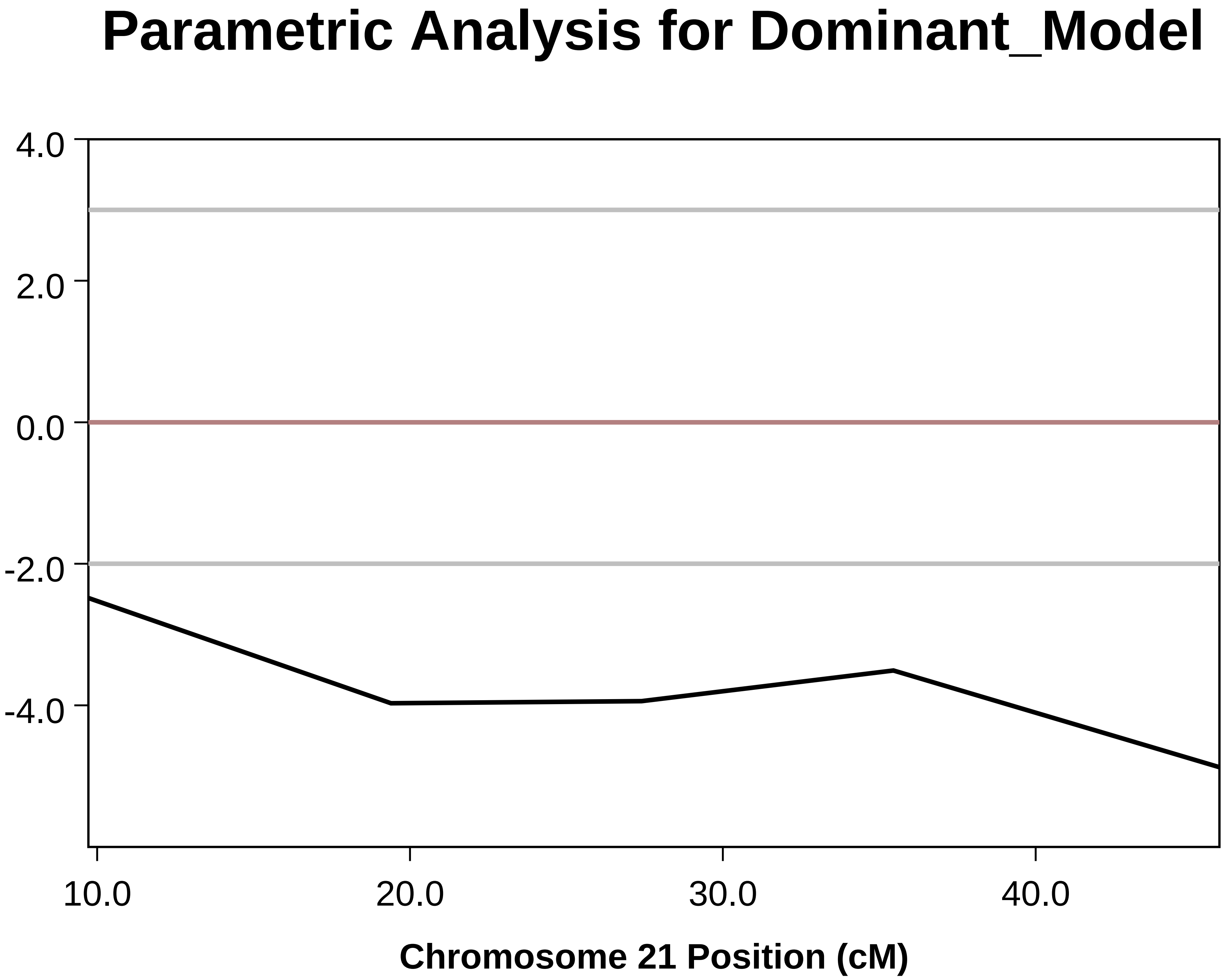
Parametric Analysis for Dominant_Model
4.0
2.0
0.0
-2.0
-4.0
10.0
20.0
30.0
40.0
Chromosome 21 Position (cM)

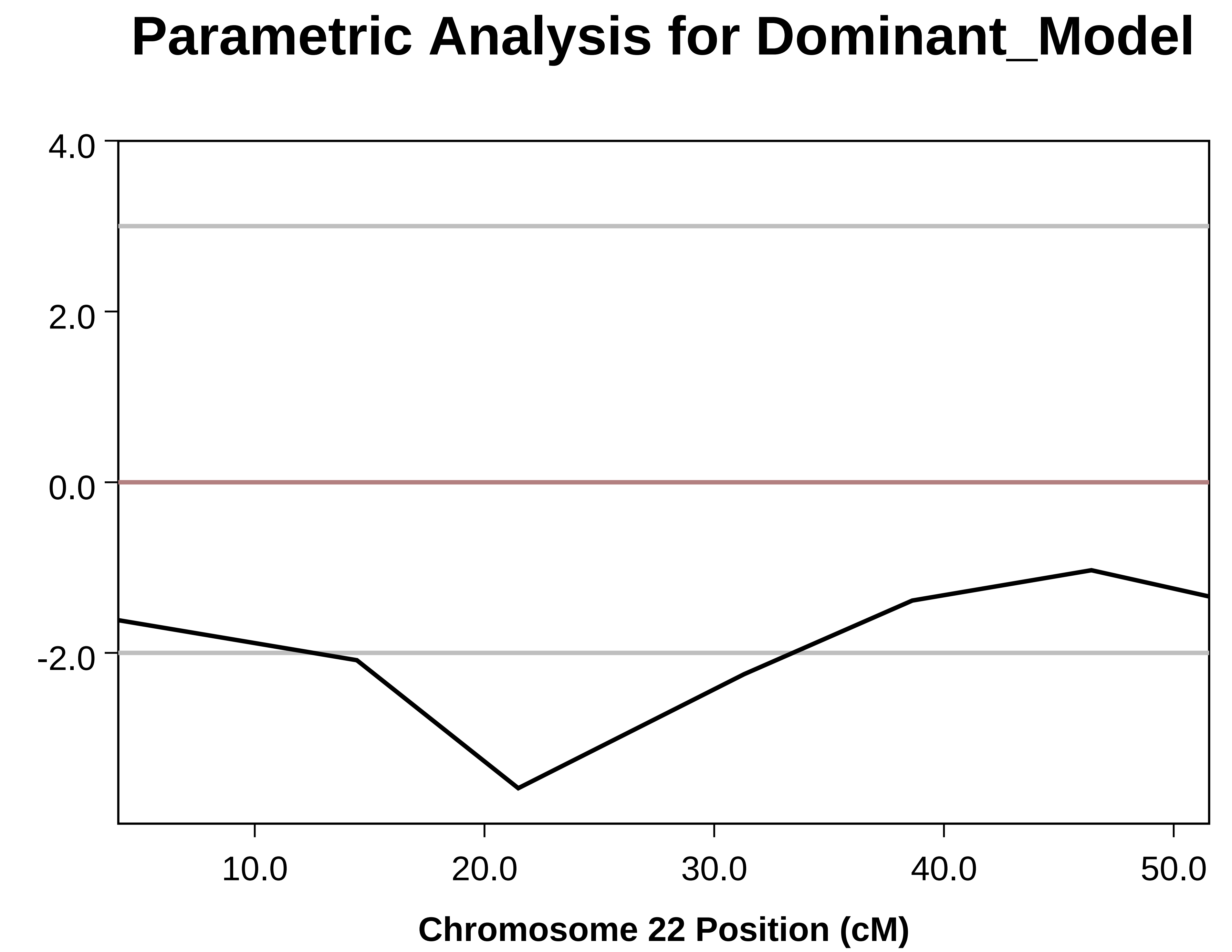
Parametric Analysis for Dominant_Model
4.0
2.0
0.0
-2.0
10.0
20.0
30.0
40.0
50.0
Chromosome 22 Position (cM)
